# Storyline description of Southern Hemisphere midlatitude circulation and precipitation response to greenhouse gas forcing

**DOI:** 10.1007/s00382-020-05234-1

**Published:** 2020-05-06

**Authors:** Julia Mindlin, Theodore G. Shepherd, Carolina S. Vera, Marisol Osman, Giuseppe Zappa, Robert W. Lee, Kevin I. Hodges

**Affiliations:** 1 Centro de Investigaciones del Mar y la Atmósfera, Consejo Nacional de Investigaciones Científicas y Técnicas, Universidad Nacional de Buenos Aires, Buenos Aires, Argentina; 2 Departamento de Ciencias de la Atmósfera y los Océanos, Facultad de Ciencias Exactas y Naturales, Universidad de Buenos Aires, Buenos Aires, Argentina; 3 Instituto Franco Argentino sobre estudios de Clima y sus impactos (IFAECI-UMI3351), Centre National de la Recherche Scientifique, Buenos Aires, Argentina; 4 Department of Meteorology, University of Reading, Reading, UK; 5 National Centre for Atmospheric Science, University of Reading, Reading, UK; 6 Istituto di Scienze dell’Atmosfera e del Clima, ISAC-CNR, Bologna, 40129 Italy

**Keywords:** Climate change, Southern Hemisphere, Storylines, Stratospheric polar vortex, Midlatitude precipitation, Atmospheric circulation

## Abstract

As evidence of climate change strengthens, knowledge of its regional implications becomes an urgent need for decision making. Current understanding of regional precipitation changes is substantially limited by our understanding of the atmospheric circulation response to climate change, which to a high degree remains uncertain. This uncertainty is reflected in the wide spread in atmospheric circulation changes projected in multimodel ensembles, which cannot be directly interpreted in a probabilistic sense. The uncertainty can instead be represented by studying a discrete set of physically plausible storylines of atmospheric circulation changes. By mining CMIP5 model output, here we take this broader perspective and develop storylines for Southern Hemisphere (SH) midlatitude circulation changes, conditioned on the degree of global-mean warming, based on the climate responses of two remote drivers: the enhanced warming of the tropical upper troposphere and the strengthening of the stratospheric polar vortex. For the three continental domains in the SH, we analyse the precipitation changes under each storyline. To allow comparison with previous studies, we also link both circulation and precipitation changes with those of the Southern Annular Mode. Our results show that the response to tropical warming leads to a strengthening of the midlatitude westerly winds, whilst the response to a delayed breakdown (for DJF) or strengthening (for JJA) of the stratospheric vortex leads to a poleward shift of the westerly winds and the storm tracks. However, the circulation response is not zonally symmetric and the regional precipitation storylines for South America, South Africa, South of Australia and New Zealand exhibit quite specific dependencies on the two remote drivers, which are not well represented by changes in the Southern Annular Mode.

## Introduction

Precipitation is a key aspect of climate, relevant for many impacts. Yet climate model projections of precipitation changes over land remain highly uncertain outside of the high latitudes (IPCC 2013). In midlatitudes, mean precipitation changes are generally dynamically rather than thermodynamically controlled (Deser et al. 2012), and the uncertainties in precipitation change are closely tied to uncertainties in changes in atmospheric circulation (Shepherd 2014; Zappa 2019).

**Fig. 1:**
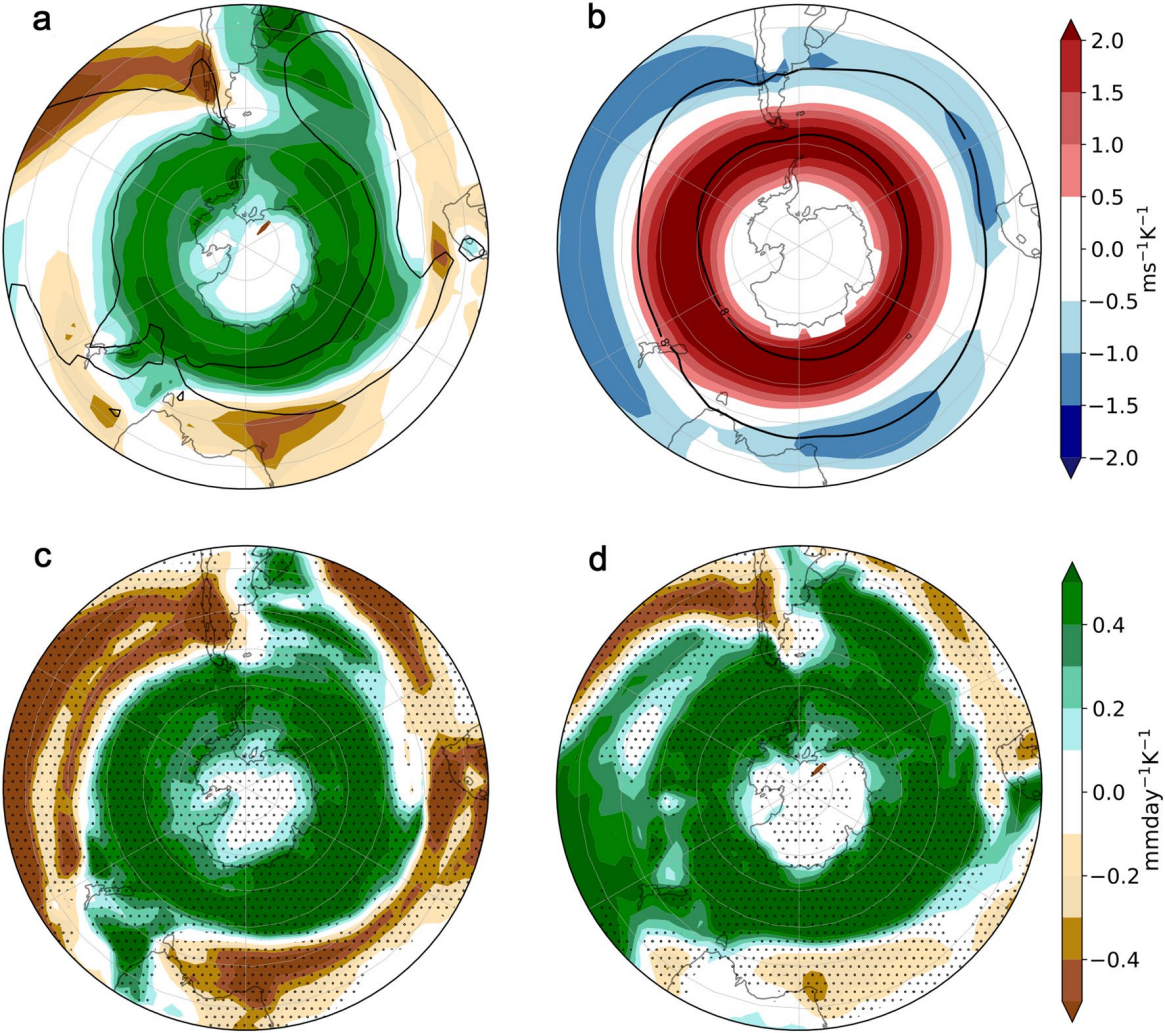
Annual mean response to climate change scaled (i.e. divided) by global warming in **a** CMIP5 multimodel ensemble mean (MEM) precipitation and **b** MEM 850-hPa zonal wind (*u*850), **c** MIROC-ESM precipitation, and **d** GFDL-ESM-2G precipitation (colours). The climate response is evaluated as the 2069–2099 mean in the RCP8.5 scenario minus the 1940–1970 mean in the historical simulations. Black contours show **a** 3 mm day$^{-1}$ and (b) $8\hbox { ms}^{-1}$ MEM climatological precipitation and *u*850 respectively in the historical simulations. The two model responses shown in panels **c**, **d** are merely to illustrate the range of model responses; they were chosen because they belong to different quadrants in the two panels in Fig. 4. Stippling in **c**, **d** indicates regions where changes are statistically significant at the $5\%$ level compared to the internal variability in each model

In the Southern Hemisphere, climate model projections show a general pattern of precipitation shift toward higher latitudes, associated with the poleward shift of the midlatitude westerlies (Scheff and Frierson 2012; illustrated in Fig. 1a, b) and storm tracks (Lee 2015). The poleward jet shift is a robust response to anthropogenic greenhouse gas forcing (Kushner et al. 2001), although the mechanisms behind it remain poorly understood (Shaw et al. 2016) and models exhibit a considerable spread in their zonal-mean response (Simpson and Polvani 2016). To illustrate the spread in the model responses, we show in Fig. 1c and d the precipitation response projected by two different models.

The uncertainties in climate model projections that are manifest in multi-model ensembles cannot be directly interpreted in a probabilistic manner (Tebaldi and Knutti 2007; Shepherd 2019). As an alternative, Zappa and Shepherd (2017) proposed a ‘storyline’ representation of the uncertainty in atmospheric circulation in terms of remote drivers of the circulation response, conditioned on global warming levels. In a storyline approach more than one physically self-consistent future evolution of global and regional climate is provided. A way of doing this is by developing the storylines so that they span the uncertainty in the future projections from multi-model ensembles. The storylines are meant to help understand the driving physical factors and their regional implications, but need not have probabilities attached to them; they are not predictions (Zappa 2019). A benefit of this approach is that it provides physically coherent descriptions of plausible changes at the regional scale, thereby allowing consideration of the correlated risk, in such a way that uncertainties at the regional scale can be reduced as knowledge about the remote driver responses improves.

The midlatitude circulation response to greenhouse gas forcing has been interpreted as a ‘tug of war’ between polar lower-tropospheric warming, which tends to shift the westerlies equatorward, and tropical upper-tropospheric warming, which tends to shift them poleward (Harvey et al. 2014; Ceppi and Shepherd 2017; Baker et al. 2017). Changes in the strength of the stratospheric vortex also contribute to the shift in the westerlies, both in the Northern (Manzini et al. 2014; Simpson et al. 2018) and in the Southern (Ceppi and Shepherd 2019) Hemispheres. Zappa and Shepherd (2017) thus used Arctic warming, tropical upper-tropospheric warming, and stratospheric vortex change, to construct storylines of European wintertime regional climate change. More recently, Garfinkel et al. (2020) has shown how such zonally averaged drivers can statistically account for a substantial portion of the spread in the annually averaged precipitation response across the midlatitudes of both hemispheres.

In this paper, we construct storylines of midlatitude climate change for the Southern Hemisphere (SH) considering tropical upper-tropospheric warming and stratospheric vortex changes as the relevant remote drivers. This is not to say that other drivers might not be important, but we ask the question: how much of the circulation response in the SH and precipitation response in the three land sectors of the midlatitude SH can be explained by these widely accepted remote drivers? We apply the Zappa and Shepherd (2017) (from now on ZS17) approach for both austral summer (December to February, DJF) and austral winter (June to August, JJA). For JJA, we also address the potential role of the jet latitude bias, as identified by Simpson and Polvani (2016), since this is potentially a confounding factor in the circulation response to the drivers.

A poleward shift of the SH midlatitude westerlies can be alternatively represented as a positive tendency of the Southern Annular Mode (SAM), coinciding with higher surface pressures in midlatitudes and lower surface pressures in high latitudes (Hartmann and Lo 1998). Indeed, there is agreement on the positive trend of the SAM as one of the most robust responses to greenhouse gas forcing (Arblaster and Meehl 2006; Arblaster et al. 2011). Enhanced precipitation in high latitudes and reduced precipitation in midlatitudes are related to the positive phase of the SAM (Silvestri and Vera 2003; Sen Gupta and England 2006). Thus, to help interpret our results in the light of previous research, we examine the projection of the circulation responses in the different storylines onto the SAM. However, it is worth noting that we regard the SAM as a (crude) description, rather than a driver, of the midlatitude circulation response.

In the satellite-era historical record, a statistically significant correlation between ENSO and the summertime SAM has been identified (L’Heureux and Thompson 2006; Silvestri and Vera 2009). Byrne et al. (2017, 2019) have argued that this correlation is mainly the result of sampling uncertainty, and that the summertime SAM variations are mainly driven by variations in the breakdown date of the stratospheric polar vortex, which happen to be correlated with ENSO in the (limited) historical record. Thus, observed correlations between SH midlatitude conditions and ENSO during the summer season may in part reflect the role of the stratosphere. It is also important to note that the zonally symmetric midlatitude circulation response to the warm conditions of El Niño appears to be opposite to the response to tropical warming under greenhouse gas forcing (Chen et al. 2008), thus one cannot interpret El Niño as a proxy for climate change. One question we address is what are the separate influences of stratospheric and tropical drivers on SH midlatitudes, in the context of climate change. Another question, given that so much literature has focused on the role of the SAM, is to what extent the midlatitude changes can be interpreted in terms of the SAM changes.

In summary, the questions we ask here are: (1) How much of the regional changes in the SH midlatitudes can be explained by the above-mentioned stratospheric and tropical drivers in the context of climate change? (2) To what extent can the midlatitude regional changes be interpreted as a result of changes in the SAM? (3) What coherent descriptions of plausible changes at the regional scale (storylines) arise based on the climate responses of the two remote drivers? (4) What is the separate and combined influence of the drivers in each storyline?

The methodology is described in Sect. 2. Austral summer and winter are treated in Sects. 3 and 4, respectively, where the target regions for analysis of precipitation changes are the regions showing a strong response in the multimodel mean. The article concludes with a Summary by region in Sect. 5 and a Discussion in Sect. 6.

## Data and methods

The methodology applied in ZS17 is used here to identify the circulation and precipitation responses to the remote drivers through linear regression of CMIP5 model projections under the RCP8.5 forcing scenario. Storm track responses are also examined in order to help link the circulation to the precipitation responses. In order to minimize the impact of the ozone hole, which has its own distinct effects on Southern Hemisphere surface climate (Thompson et al. 2011), we consider the difference between the time periods 1940–1970 of the historical simulation and 2069–2099 of the RCP8.5 simulation (Taylor et al. 2012). This excludes the period in between, where ozone depletion has a discernible impact on the Antarctic vortex in climate model simulations (McLandress et al. 2010). We invoke the pattern scaling assumption (Tebaldi and Arblaster 2014) and scale the individual responses by the model’s global-mean warming (i.e., we divide by the global-mean warming), in order to remove global-mean warming as a confounding factor in the regression. Pattern scaling is a reasonable assumption here since we are considering the different models under the same (transient) radiative forcing and the same time horizon (Ceppi et al. 2018).

### CMIP data

We used data from 32 CMIP5 models. The primary fields of interest are the zonal wind *u* at 850 hPa (*u*850) and precipitation, although we have also analyzed sea level pressure and cyclone density. The cyclone density was computed using the TRACK algorithm, the same method as was used in Hoskins and Hodges (2002) and reproduced by Lee (2015). The algorithm identifies cyclones in the 6 hourly 850hPa relative vorticity field and groups them into trajectories using a constrained minimization of a cost function for the ensemble track smoothness to obtain the minimal set of smoothest tracks. The track density is computed from these tracks using spherical kernel estimators (Hodges 1996) and subsequently scaled to number density per month per unit area where the unit area is equivalent to a 5 degree spherical cap ($\approx \, 10^{6}\hbox { km}^{2}$). Because 6-hourly data is required, and this data is only available from 1950, the 1950–1980 climatology of the historical simulation was used to define the response of that field. The future period for the storm track analysis was the same as for all other fields. All model data was regridded to a common T42 spatial grid using bilinear interpolation for all variables except precipitation, for which we used conservative remapping. For models that provided more than one ensemble member we computed ensemble means using all available ensemble members that share the same physics (r$\#$i1p1). In Table 1 we show the details of the models used for the study.

**Table 1:** List of CMIP5 models considered in the study. Resolutions are shown in degrees ( lat $\times$  lon)

Basic information			No. monthly runs		No. daily runs	
	Model name	Resolution	Historical	RCP 8.5	Historical	RCP 8.5
1	ACCESS1.0	1.25 $\times$ 1.875	1	1	1	1
2	ACCESS1.3	1.25 $\times$ 1.875	3	1	1	1
3	BCC-CSM11	2.7906 $\times$ 2.8125	3	1	1	1
4	BCC-CSM11m	2.7906 $\times$ 2.8125	3	1	1	1
5	BNU-ESM	2.7906 $\times$ 2.8125	1	1	1	1
6	CCSM4	0.9424 $\times$ 1.25	6	6	1	1
7	CESM1(CAM5)	0.9424 $\times$ 1.25	3	3	–	–
8	CMCC-CM	0.7484 $\times$ 0.75	1	1	1	1
9	CMCC-CMS	3.7111 $\times$ 3.75	1	1	1	1
10	CMCC-CESM	3.4431 $\times$ 3.75	1	1	1	1
11	CNRM-CM5	1.4008 $\times$ 1.40625	10	10	1	1
12	CSIRO Mk3.6.0	1.8653 $\times$ 1.875	10	10	1	1
13	CanESM2	2.7906 $\times$ 2.8125	5	5	5	5
14	EC-EARTH	1.1215 $\times$ 1.125	2	2	2	2
15	FIO-ESM	2.8125 $\times$ 2.789327	3	5	–	–
16	GFDL CM3	2 $\times$ 2.5	5	1	3	1
17	GFDL-ESM2G	2.0225 $\times$ 2	1	1	1	1
18	GFDL-ESM2M	2.0225 $\times$ 2.5	1	1	1	1
19	GISS-E2-H	2 $\times$ 2.5	2	2	–	–
20	GISS-E2-R	2 $\times$ 2.5	2	2	–	–
21	HadGEM2-CC	1.25 $\times$ 1.875	3	3	1	1
22	INM-CM4	1.5 $\times$ 2	1	1	1	1
23	IPSL-CM5A-LR	1.8947 $\times$ 3.75	5	4	3	3
24	IPSL-CM5A-MR	1.2676 $\times$ 2.5	3	1	3	1
25	IPSL-CM5B-LR	1.8947 $\times$ 3.75	1	1	1	1
26	MIROC-ESM	2.7906 $\times$ 2.8125	3	1	3	1
27	MIROC-ESM-CHEM	2.7906 $\times$ 2.8125	1	1	1	1
28	MIROC5	1.4008 $\times$ 1.40625	5	3	5	3
29	MPI-ESM-LR	1.8653 $\times$ 1.875	3	3	3	3
30	MPI-ESM-MR	1.8653 $\times$ 1.875	3	1	3	1
31	MRI-CGCM3	1.12148 $\times$ 1.125	3	1	1	1
32	NorESM1-M	1.8947 $\times$ 2.5	3	1	3	1

For each model, the number of ensemble members for which monthly and daily data are available are indicated for the historical and RCP8.5 simulations. The dash indicates that daily data are not available

For the DJF analysis we used monthly mean fields of surface air temperature and temperature at 250 hPa, and daily zonal wind at 50 hPa, to build the indices describing the remote drivers (defined in Sect. 2.2). Because daily data was needed to compute the vortex breakdown date, only models providing daily data were used for this season (see Table 1). For the JJA analysis, we used monthly mean fields of surface air temperature, temperature at 250 hPa, and zonal wind at 50 hPa to build the driver indices. For the analysis of the model bias in the latitude of the jet in the reference climatological period (described in Sect. 4.1), the latitude of the jet was defined as the centroid of the 850-hPa zonal wind distribution between $30^{\circ }$ and $70^{\circ }\hbox {S}$:

1 $$\begin{aligned} \bar{\lambda } = \frac{\int _{-70}^{-30} \lambda [u(\lambda )]^{2} d\lambda }{\int _{-70}^{-30} [u(\lambda )]^{2} d\lambda } \end{aligned}$$

where $\bar{\lambda }$ is the jet latitude, $[u(\lambda )]$ is the zonal mean zonal wind, and easterlies (i.e., negative values of $[u(\lambda )]$) were excluded from the calculation. This jet definition was used in Ceppi et al. (2018).

### Definition of remote drivers

Manzini et al. (2014) and ZS17 showed how indices that capture intermodel spread in the climate change projections can contribute to explain part of the uncertainty in tropospheric circulation changes in the Northern Hemisphere. We made a similar assessment to identify remote drivers of the austral midlatitude circulation response to greenhouse gas forcing and the associated global warming. We analyzed the intermodel spread in the temperature and wind responses to global warming (not shown) and found temperature at around 250 hPa as one of the aspects of climate with the largest uncertainty in both DJF and JJA. It has been established that tropical upper-tropospheric warming can induce a midlatitude circulation response (Butler et al. 2010; Arblaster et al. 2011). We therefore defined a tropical warming index ($\varDelta \,T_{trop}$) based on the change in temperature at 250 hPa zonally averaged between $15^{\circ }\hbox {S}$ and $15^{\circ }\hbox {N}$. During JJA, the stratospheric zonal wind above 60 hPa between $50^{\circ }\hbox {S}$ and $60^{\circ }\hbox {S}$ emerges as a potential source of uncertainty, together with lower stratospheric temperature between $60^{\circ }\hbox {S}$ and $90^{\circ }\hbox {S}$. Since these two features are related, we describe this stratospheric source of uncertainty in JJA using a single index ($\varDelta \, U_{strat}$), defined as the zonal wind changes at 50 hPa, zonally averaged between $50^{\circ }\hbox {S}$ and $60^{\circ }\hbox {S}$. Although there is no vortex during the warm season, changes in the strength and persistence of the stratospheric vortex during the preceding spring contribute to a shift of the summer westerlies. Previous work has shown a time-lagged influence of the spring stratospheric vortex on the tropospheric zonal winds in DJF on both sub-seasonal and seasonal time-scales (Mechoso et al. 1988; Thompson and Wallace 2000; Saggioro and Shepherd 2019) and in the forced response (Ceppi and Shepherd 2019). There is agreement across models on a delayed vortex breakdown in the future climate under the RCP8.5 scenario, but with delays varying from 5 to more than 30 days (Ceppi and Shepherd 2019), representing another source of uncertainty. Thus, to describe the influence of the stratosphere in DJF, we defined a stratospheric vortex breakdown delay index ($VB_{delay}$) as the difference between the climatological vortex breakdown date in the future period and the climatological breakdown date in the reference period. The vortex breakdown date is defined as the time when the polar vortex first weakens below 15 $ms^{-1}$ in its seasonal march (Ceppi and Shepherd 2019), in units of Julian days. Summarizing, the driver indices considered are the following:

- DJF and JJA: Tropical upper-tropospheric warming ($\varDelta \,T_{trop}$)
- JJA: Stratospheric vortex strengthening ($\varDelta \, U_{strat}$)
- DJF: Stratospheric vortex breakdown delay ($VB_{delay}$)

The global warming index ($\varDelta \,T$) is computed as the global average of the annual mean change of surface air temperature. All spatial averages are area weighted.

**Fig. 2:**
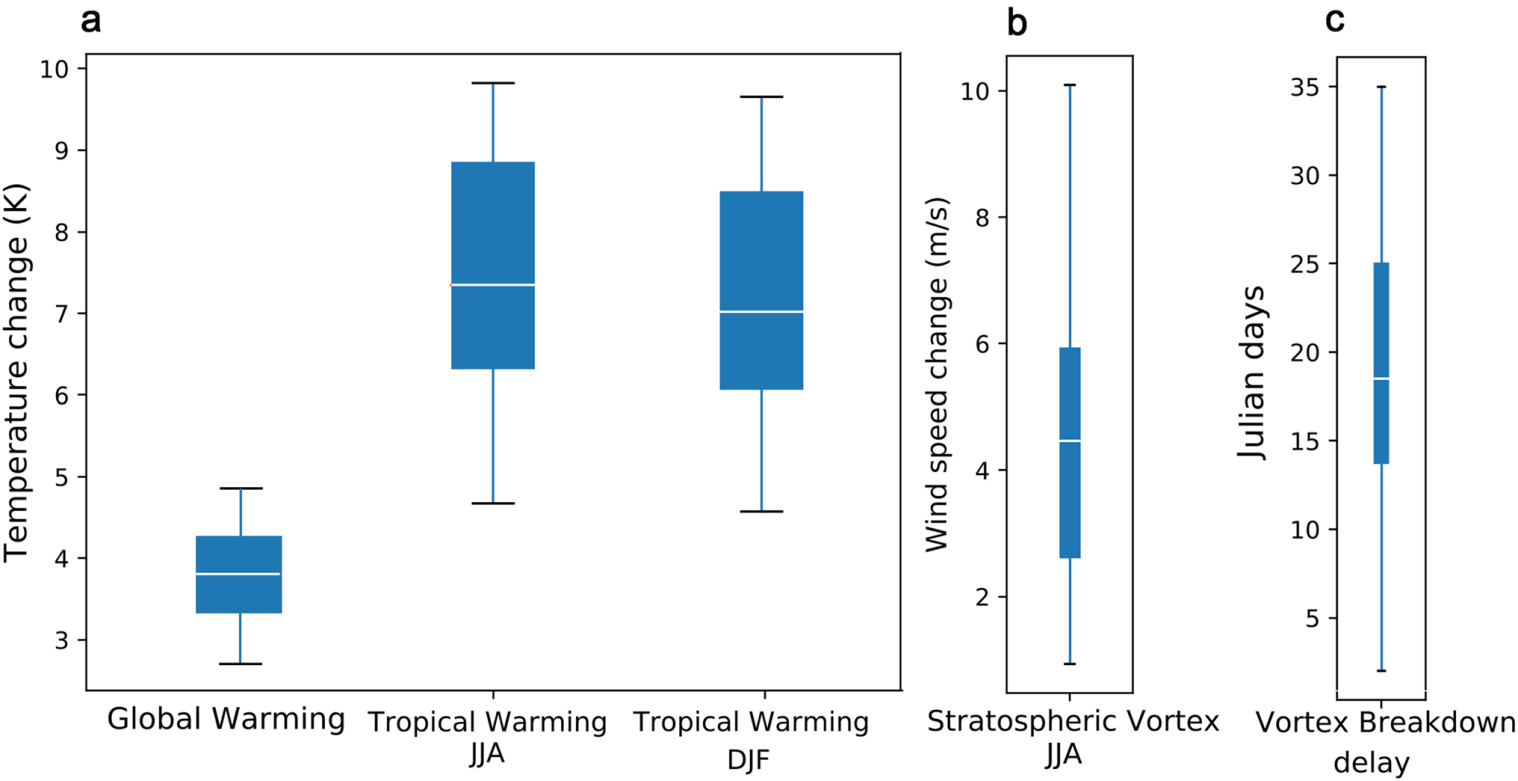
Spread among the climate change responses for the CMIP5 model ensemble for 2069–2099 in the RCP8.5 scenario minus 1940–1970 in the historical simulation. **a** Global surface warming (global warming, $\varDelta T$) and 250-hPa warming over $15^{\circ }\hbox {S}$-$15^{\circ }\hbox {N}$ (tropical warming, $\varDelta T_{trop}$), **b** 50-hPa zonal wind change over $50^{\circ }$-$60^{\circ }\hbox {S}$ (stratospheric vortex strengthening, $\varDelta U_{strat}$), **c** vortex breakdown delay ($VB_{delay}$). Global warming is evaluated for the annual mean, the tropical warming is evaluated for each season, vortex strengthening is evaluated in JJA, and the vortex breakdown delay takes place between October and December. The box plots show the multimodel ensemble median (white line), the lower and upper quartiles (box) and the full range (whiskers)

Similarly to ZS17, we defined the remote drivers as the indices defined above scaled by the global warming index (i.e., divided by the global-mean warming in each model). We refer to them in the text as tropical warming (TW), vortex strengthening (VS) and vortex breakdown (VB) delay. We refer to their extreme values within the CMIP5 ensemble as “High/Low TW”, “Large/Small VS” and “Late/Early VB” respectively. The response of each index (i.e. the remote drivers without scaling by global warming) is shown in Fig. 2 for both seasons. The models agree on the sign of the strengthening of the stratospheric vortex and in the enhanced warming in the tropical upper troposphere. There is a correlation of 0.36 (p-value 0.06) between the vortex breakdown delay and the tropical warming before scaling by global warming, but it becomes insignificant after scaling by global warming (Pearson correlation coef.: 0.14; p-value: 0.47). The correlation between the JJA indices before scaling by global warming is 0.51 (p-value: 0.002), which after scaling becomes 0.27 (p-value: 0.07). We analyzed the statistical significance of the correlation between the tropical upper-tropospheric temperature and the stratospheric vortex strength in the interannual variability during the winter season (June–July–August) using data from the ERA-Interim reanalysis (Dee et al. 2011). The indices were defined in correspondence with the indices of the main study:

- Upper-tropospheric tropical temperature ($T_{trop}$): temperature at 250 hPa zonally averaged between $15^{\circ }\hbox {S}$ and $15^{\circ }\hbox {N}$
- Stratospheric vortex strength ($U_{strat}$): zonal wind at 50hPa zonally averaged between $50^{\circ }\hbox {S}$ and $60^{\circ }\hbox {S}$

**Fig. 3:**
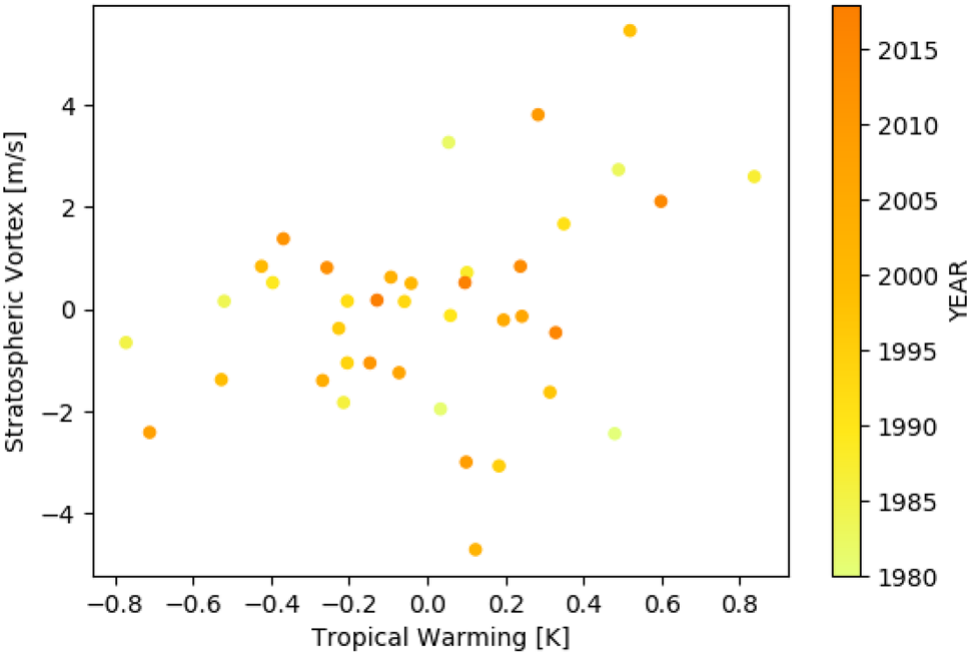
Interannual variability of the observed upper-tropospheric temperature and stratospheric vortex strength during the winter season (June–July–August) for the period 1980–2018. Pearson correlation: 0.33 (p-value: 0.03)

The interannual variation of the detrended indices is shown in Fig. 3. The Pearson correlation between the detrended indices is 0.33 (p-value: 0.03).

### Regression framework

Pattern scaling is commonly implemented by computing a spatial map of the changes in a variable for a certain model ($\varDelta C_{xm}$), defined usually as the difference between two multi-decadal averages, and normalizing them by the change in global average temperature of the corresponding model ($\varDelta T_{m}$; Tebaldi and Arblaster 2014). Applying this scaling, the climate response patterns take the form

2 $$\begin{aligned} \varDelta C_{xm} = \varDelta T_{m} P_{xm}, \end{aligned}$$

where $P_{xm}$ is the pattern of the climate response at grid point *x* of model *m*. Applying the pattern scaling assumption was one of the key innovations of ZS17. This enabled the separation of the uncertainty in the pattern of the response from the uncertainty in the global warming level. Possible limitations of this approach are discussed in Sect. 6. This separation is useful because it is reasonable to assume that the patterns of change are affected by different sources of model uncertainty, other than global warming itself. Also, it eliminates the different climate sensitivities of the models as a potential confounding factor in the regression analysis. As in ZS17, after applying pattern scaling we express the regional response as a linear combination of the responses to the two remote drivers (indices scaled by global warming). The linear models for the DJF and JJA seasons are given by:

*DJF linear model*

3 $$\begin{aligned} P_{xm} = a_{x} + b_{x} \left( \frac{\varDelta T_{trop}}{\varDelta T}\right) _{m}' + c_{x} \left( \frac{VB_{delay}}{\varDelta T}\right) _{m}' + e_{xm}. \end{aligned}$$

*JJA linear model*

4 $$\begin{aligned} P_{xm} = a_{x} + b_{x} \left( \frac{\varDelta T_{trop}}{\varDelta T}\right) _{m}' + c_{x} \left( \frac{\varDelta U_{strat}}{\varDelta T}\right) _{m}'+ e_{xm}. \end{aligned}$$

Here the $'$ indicates the standardized anomaly with respect to the multimodel mean. $a_{x}$ represents the multimodel ensemble mean (MEM) response per degree of global warming. In the DJF model, the coefficients $b_{x}$ and $c_{x}$ quantify the sensitivity of the regional response to the uncertainties in the remote drivers $\varDelta T_{trop}/\varDelta T$ and $VB_{delay}/\varDelta T$ respectively, and their estimated values $\hat{b}_{x}$ and $\hat{c}_{x}$ are computed by fitting the model (3) to CMIP5 data using ordinary multiple linear regression. In JJA, the coefficients $b_{x}$ and $c_{x}$ quantify the sensitivity of the regional response to the uncertainties in the remote drivers $\varDelta T_{trop}/\varDelta T$ and $\varDelta U_{strat}/\varDelta T$ respectively. However, as mentioned above, the TW and VS drivers exhibit a weak correlation, which is also present in the ERA-Interim reanalysis inter-annual variability (Fig. 3). We therefore need to allow for the possibility that there is a physical connection between the changes in the tropics and in the stratosphere. Thus we cannot apply simple multiple linear regression, instead we do sequential regressions as in Manzini et al. (2014) to compute the sensitivities to the remote drivers (see “Appendix A” for mathematical details). Applying a linear regression approach implies assuming independent and identically distributed residuals $e_{xm}$. This is not the case for CMIP5 data (Knutti et al. 2013) and because of this, the correlations across models have to be considered with caution because of shared biases.

**Fig. 4:**
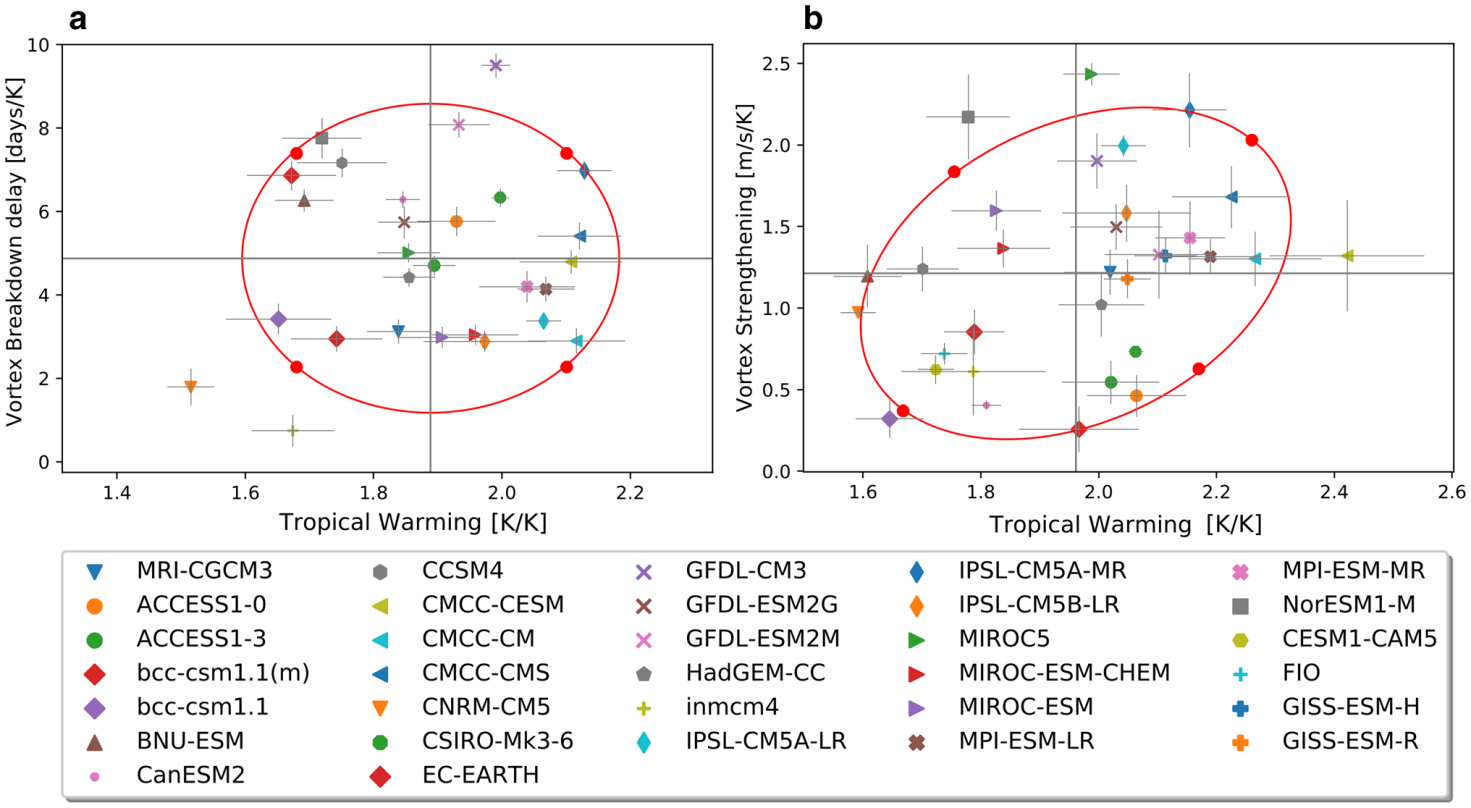
CMIP5 model responses in **a** VB delay and TW in DJF, and in **b** VS and TW in JJA. The red curve shows the 80 $\%$ confidence ellipse of the joint $\chi ^{2}$ distribution with two degrees of freedom. The red dots in **a** and **b** indicate the storylines defined for DJF and JJA respectively. The DJF storylines are equally distant from the MEM driver responses (grey lines), but the JJA storylines are not equally distant due to the correlation between the two drivers. Error bars show the $95\%$ confidence interval in the individual model responses of $\varDelta T_{trop}/\varDelta T$, $\varDelta U_{strat}/\varDelta T$ and $VB_{delay}/\varDelta T$. The confidence intervals are estimated, assuming white noise, from the year-to-year variability in the remote drivers, and also accounting for the number of ensemble members available for each model (see “Appendix B”)

### Storyline evaluation

In order to generate a diverse set of plausible storylines of the tropospheric midlatitude climate response to greenhouse gas forcing, we evaluate each field as the combination of its multi-model mean response with the sensitivities to the remote drivers (coefficients in Eq. 3 for DJF and Eq. 4 for JJA). Figure 4 shows the range of remote driver responses in the CMIP5 ensemble and how the storylines (represented by the red dots) are chosen such that they represent responses of the remote drivers with equal standardized anomaly amplitudes. To generate extreme but plausible storylines, they are chosen to lie on the edge of the 80$\%$ confidence region of the joint distribution as in ZS17. The storylines show a climate response per degree of warming conditioned on the response of the remote drivers. Each storyline is characterized by a combination of high or low TW and either large or small VS (JJA) or late or early VB delay (DJF) compared to the MEM. For each storyline, we compute the SAM response as the difference between the seasonal zonally-averaged sea level pressure response at $40^{\circ }\hbox {S}$ and $65^{\circ }\hbox {S}$ as in Lim et al. (2016), who adapted the definition of Gong and Wang (1999) for application to a climate change assessment. A similar SAM index, except averaged over one month instead of three months as in our case, was also used by Marshall (2003) to address SAM trends. To test the robustness of the results, we evaluated the storylines by averaging together the circulation response, scaled by global-mean surface warming, of models that have similar driver responses (not shown). For each season, models were grouped within the four quadrants of Fig. 4a, b.

## DJF

### Circulation and precipitation sensitivity to remote drivers

**Fig. 5:**
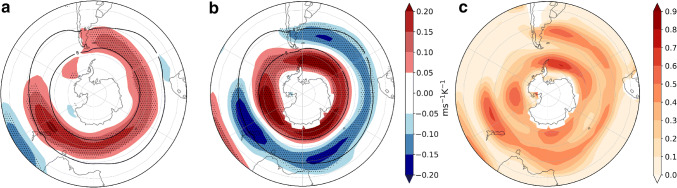
Sensitivities of the circulation response associated with the uncertainties in the remote driver responses in DJF determined using the multiple linear regression model (3). **a***u*850 response scaled by global warming associated with one standard deviation positive anomaly in the TW ($\varDelta T_{trop}/\varDelta T$) in the CMIP5 model ensemble spread. Stippling indicates areas with regression coefficients statistically significant at the $5\%$ level, evaluated with a two-tailed* t*-test. Black contours show the $8\hbox { ms}^{-1}$ MEM *u*850 in the historical simulations. **b** As **a** but uncertainty associated with the VB delay ($VB_{delay}/\varDelta T$) and **c** fraction of variance ($R^{2}$ coefficient) explained by the linear model (3)

We analyzed the circulation response to the remote drivers introduced in Sect. 2.2 by applying the regression framework in Sect. 2.3 to *u*850. The climatological SH zonal winds have a fairly symmetric structure in DJF, although the westerly winds centered at $45^{\circ }\hbox {S}$ are slightly stronger eastward of South America and across the South Atlantic and Indian oceans, and are weaker in the South Pacific. As was mentioned in the Introduction, wind variability is partially described by the SAM index. When the latter is in its positive phase, the band of westerly winds strengthens and moves poleward. However, the responses of the winds to TW and to the delay in the VB are very different in their spatial structure and magnitude (Fig. 5). The magnitude of the flow response is influenced by the magnitude of the uncertainty in the driver response, as well as the strength of the teleconnection. The response to TW is characterized by a strengthening of the westerly winds to the east of South America and a marked strengthening over New Zealand without a significant meridional shift (Fig. 5a). On the other hand, the response to the VB delay is associated with a clear poleward shift of the westerly winds, with a highly symmetric structure, and it bears a remarkable resemblance to the wind anomaly structure associated with the positive phase of the SAM (Fig. 1d in Sen Gupta and England (2006)). The response pattern also exhibits a wave-3 structure with anticyclonic circulation anomalies east of South America and both east and west of Australasia (Fig. 5b). A similar wave-3 structure has been previously associated with the SAM by Fogt et al. (2012). These results agree with Ceppi and Shepherd (2019), who identified a poleward shift of the zonal mean jet as a response to the delay in the vortex breakdown induced by greenhouse gas forcing. Figure 5c shows that the two drivers explain locally up to $70{-}80\%$ of the inter-model variance.

**Fig. 6:**
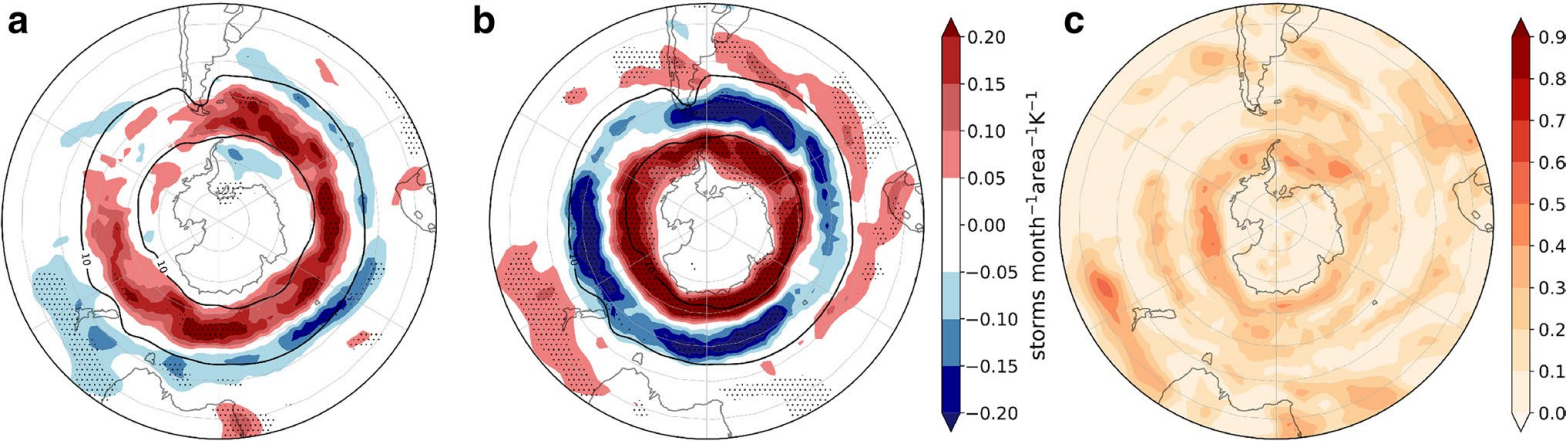
As Fig. 5 but for cyclone density (storms month$^{-1}$ unit area$^{-1}$ K^−1^; the unit area is equivalent to a $5^{\circ }$ spherical cap $\approx \, 10^{6}\hbox { km}^{2}$). Black contours show the 10 storms month$^{-1}$ unit area$^{-1}$ MEM in the historical simulation

To interpret the impact of the westerly wind changes on precipitation we also assessed the sensitivity of the cyclone density (defined in Sect. 2.1 as the number of cyclones per month per unit area with a unit area equivalent to $$10^{6}$$$$\hbox {km}^{2}$$) to the remote drivers (Fig. 6). South of $$50^{\circ }\hbox {S}$$ the zonally asymmetric response to both drivers is consistent with the response of the zonal winds. The cyclone density response is increased in the position of the climatological-mean storm-track maximum in association with the TW, and the strongest response is located in the South Atlantic (Fig. 6a). On the other hand, the cyclone density is increased on the poleward side of the climatological storm track in response to the VB delay, which is consistent with the circulation response. Furthermore, a cyclone density increase is also discernible on the equatorward side of the climatological mean cyclone density maximum. The locations of the maximum poleward shifts of the cyclone density are collocated with the wave-3 pattern observed in the *u*850 response (Fig. 6b). North of $$50^{\circ }\hbox {S}$$ there is a cyclone density increase in response to the VB delay, maximized over South Africa and the east coast of South America and Australia. Fig. 6c shows that the two drivers explain locally up to 50–60% of the inter-model variance.

**Fig. 7:**
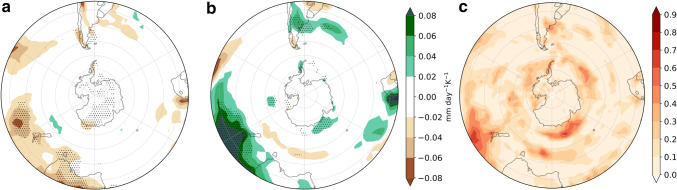
As Fig. 5 but for precipitation response (mm day$^{-1}\hbox { K}^{-1}$)

Lastly, we examine the explanatory power of the two remote drivers for the precipitation response (Fig. 7). Where the responses are statistically significant, TW is mainly related to drying (Fig. 7a) and VB delay to wetting (Fig. 7b). The drying response to TW is centered at $45^{\circ }\hbox {S}$, consistent with the diminishing of cyclone density related to this remote driver. Also, wetting on the west coasts of the continents as a response to the VB delay can be related to the enhanced cyclone density in these same regions. In the next section we analyze the storylines related to the extreme responses of the two drivers and concentrate on inhabited regions because of the socio-economic impact that precipitation changes might induce, therefore we do not analyze the Antarctic coast. However, we remark that this is one of the regions where both drivers have explanatory power. The fraction of variance explained by the linear model locally reaches $60\%$, but is generally lower for this field than for the other fields. However, agreement with the circulation and cyclone density responses provides robustness to the results.

**Fig. 8:**
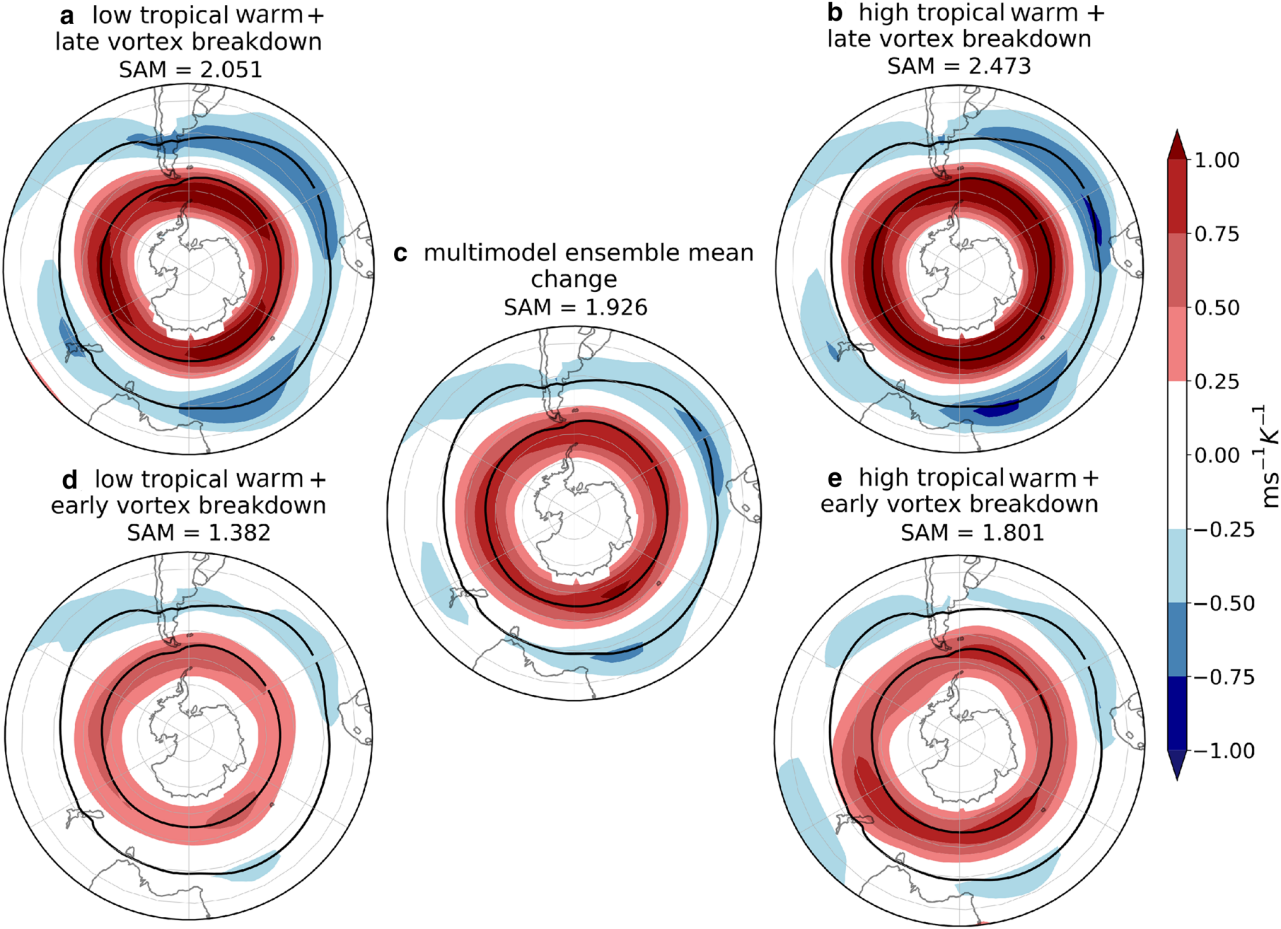
DJF *u*850 response per degree of warming ($\hbox {ms}^{-1}\,\hbox {K}^{-1}$), meaning that to obtain the response for a global-mean warming of $2^{\circ }\hbox {C}$ these values should be multiplied by two. **a**, **b**, **d**, **e** are plausible storylines of climate change related to extreme values of TW and VB delay. **c** shows the MEM *u*850 response. Black contours show the $8\hbox { ms}^{-1}$ MEM *u*850 in the historical simulations. SAM index (hPa $\,\hbox {K}^{-1}$) is computed as the change in the mean climatological SAM

**Fig. 9:**
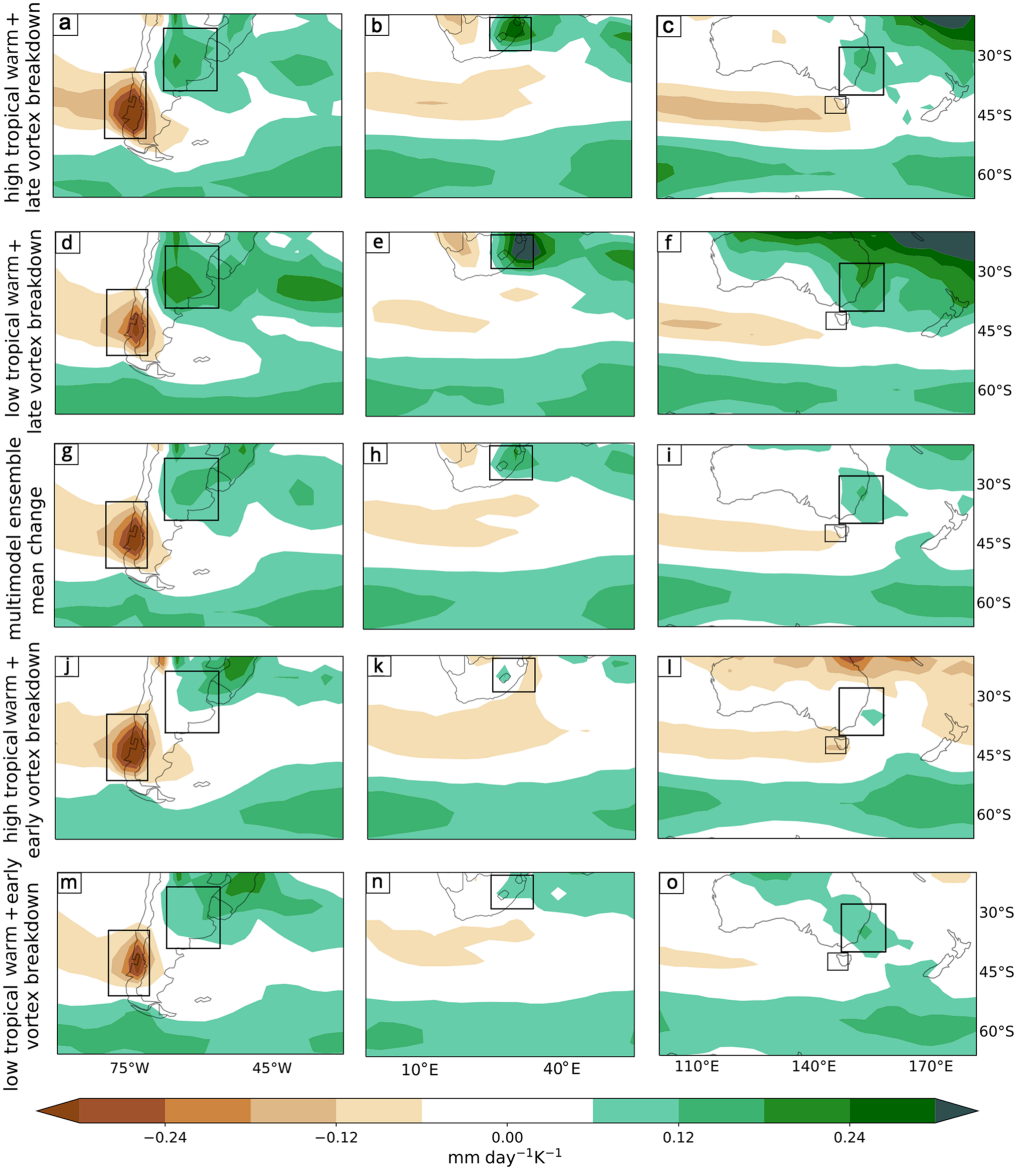
DJF precipitation response per degree of warming (mm day$^{-1}K^{-1}$) in midlatitude regions of **a** South America, **b** South Africa and **c** Australasia for the “High TW - Late VB” storyline (Fig. 8b). The same for the “Low TW - Late VB” storyline (Fig. 8a) is shown in **d**, **e** and **f**, the “High TW - Early VB” storyline (Fig. 8e) in **j**, **k** and **l** and the “Low TW - Early VB” storyline (Fig. 8d) in **m**, **n** and **o**. The MEM response is shown in **g**, **h** and **i**

### Storylines of regional wind and precipitation changes

We constructed four storylines of climate change corresponding to extreme states of the remote drivers for DJF (see mathematical details in “Appendix A”), in addition to the MEM. Hemispheric maps for *u*850 changes in each storyline (Fig. 8) and selected domain maps for precipitation (Fig. 9) are explained in this section. We computed a SAM index for each storyline (as explained in Sect. 2.4) as a quantification of the zonal-mean circulation change. The variability of the SAM has been widely studied in its relationship with that of the precipitation anomalies (Silvestri and Vera 2003; Sen Gupta and England 2006; Silvestri and Vera 2009). In addition, the impact of the projected SAM trend on future precipitation changes in the SH has also been identified (Lim et al. 2016). Therefore the SAM response associated with each of the storylines was also estimated to complement the interpretation of the precipitation response in each storyline.

Figure 8 shows the *u*850 response maps for the four storylines considered, as well as the MEM. A storyline with high TW ($\sim \, 2.1\hbox { KK}^{-1}$) and comparatively late VB ($\sim \, 7.5\hbox { day K}^{-1}$; Fig. 4a, upper right) is associated with a strengthening and poleward shift of the westerly winds across the hemisphere and easterly anomalies to the west of South Africa and Australia (Fig. 8b). The opposite storyline, with a low TW ($\sim \, 1.65\hbox { KK}^{-1}$) and comparatively early VB ($\sim 2.5\hbox { day K}^{-1}$; Fig. 4a, lower left) is associated with a weak annular circulation response (Fig. 8d). Inspection of the two intermediate storylines indicates that both storylines with a late VB show a much stronger response compared to the early VB storylines, indicating a dominant influence of the stratospheric VB uncertainty over the TW uncertainty in this season. The storylines in Fig. 8b, d are the most extreme in terms of the SAM response while those in Fig. 8a, e have SAM responses not too different from the MEM, even though their patterns feature some substantial regional differences. This indicates that the SAM response is only a crude descriptor of the regional circulation response in this season.

We assessed the precipitation changes in the vicinity of the three continental domains at midlatitudes ($30^{\circ }\hbox {S}$–$60^{\circ }\hbox {S}$). The precipitation changes associated with the four storylines in Fig. 8 are shown in Fig. 9. The storylines in Fig. 9a–c and m–o are related to the circulation changes shown in Fig 8b and d respectively, which are associated with the extreme values of the SAM index. The storylines in Fig. 9d–f and j–l are related to the intermediate storylines in terms of the SAM index (Fig. 8a and e respectively). Across the domains we identify five regions that show a strong signal in the multimodel ensemble mean (Fig. 9g–i). (Although there could in principle be regions with a strong response to the storylines but a weak response in the MEM, we did not find any such regions here, nor in JJA.) Table 2 shows, for each region, the area average contribution of each remote driver to the precipitation change per degree of warming and the precipitation change per degree of warming for all four storylines together with the median absolute deviation of the area averaged residuals of the linear model (3). The latter is included as an indication of the noise level in the analysis. The ordering of the area average precipitation changes between storylines is the same if the storylines are instead evaluated through model averages (Sect. 2.4), which provides a measure of robustness (not shown). The general pattern of change is characterized by a wetting on the eastern side of the continents at subtropical latitudes extending eastwards, and drying on the western side in the midlatitudes extending towards the west.

**Table 2:** Area average of DJF precipitation changes (mm day $^{-1}\hbox { K}^{-1}$) associated with each remote driver, and in the four storylines shown in Fig. 9, together with the median absolute deviation (MAD) of the residuals from the statistical model (Eq. 3)

DJF							
Region	TW	VB	Low TW Early VB	High TW Early VB	Low TW Late VB	High TW Late VB	Residual MAD
Extratropical Andes	− 0.019	− 0.004	− 0.11	− 0.16	− 0.12	− 0.17	0.02
Southeastern South America	− 0.016	0.029	0.08	0.04	0.16	0.12	0.06
East of South Africa	− 0.024	0.062	0.04	− 0.02	0.20	0.14	0.12
South East of Australia	− 0.026	0.027	0.07	0.00	0.14	0.07	0.03
Tasmania	− 0.033	0.012	− 0.03	− 0.11	0.00	− 0.08	0.04

While the precipitation response to greenhouse gas forcing has been shown to lead to an overall increase of tropical precipitation, a reduction of precipitation in midlatitudes (drying band) and increased precipitation in high latitudes, there is a strong seasonality to this change (IPCC 2013). Lim et al. (2016) shows that in the SH the drying band associated with greenhouse gas forcing is located more poleward in the warm season than in the cold season, which is associated with a greater poleward shift of the westerlies and the storm tracks in the warm season, compared to the cold season. Thus, the poleward shift of the storm tracks (Fig. 8) can explain the drying over the continental regions (defined in Table 2) that are located further south, which are the Extratropical Andes and Tasmania. In both regions the drying is mainly affected by TW.

The subtropical east coasts of the three continental regions experience future DJF precipitation increases related to precipitation changes in the South Atlantic Convergence Zone (Southeastern South America), South Indian Convergence Zone (South East of South Africa) and South Pacific Convergence Zone (South East of Australia). This means that precipitation changes cannot be interpreted solely in relation to changes in the westerly winds and storm tracks. In these three regions both drivers are important, but the TW acts in the opposite sense to the VB delay (Table 2). For the same VB response, the storylines show drier conditions if the TW is high, while for the same TW response the storylines project more wetting when the VB is delayed. This means that the strongest wetting arises from the “Low TW-Late VB” storyline, whereas the “High TW-Early VB” storyline has almost no wetting (Table 2, Fig. 9d–f and j–l). Thus the storylines related to extreme values of the SAM index are not the most extreme storylines for these regions. In all three subtropical regions, wetting is associated with an enhanced cyclone density, which responds strongly to the VB delay (Fig. 6b); this is also seen in Fig. 7b.

Overall, in DJF, high TW generally leads to drying and delayed VB to wetting, but the sensitivity to each driver has a strong regional dependence. In the midlatitude regions the wetting from delayed VB is opposed to some extent by the drying from TW. Since the SAM index is approximately equally sensitive to both remote drivers, with the same sign of response, this shows that the DJF regional precipitation changes over land are not at all well characterized by the SAM response.

## JJA

For this season, we first addressed the potential role of the biased jet latitude in the models as a confounding factor for the regression analysis. To do this we analyzed the correlation between the climatological jet position in the historical simulations and the remote drivers defined for JJA (Sect. 2.2).

**Table 3:** Pearson correlation coefficients between (upper row) the climatological jet position ($\phi _{0}$) in the historical period (1940–1970) and different indices of climate change: global warming (GW), and the response of the remote drivers (tropical warming and vortex strengthening) of JJA circulation (evaluated as in Sect. 2.3), with (TW, VS) and without ($\varDelta \hbox {T}_{trop}$, $\varDelta \hbox {U}_{strat}$) scaling by GW

	GW	$\varDelta \hbox {T}_{trop}$	TW	$\varDelta \hbox {U}_{strat}$	VS
$\phi _{0}$ w IPSL	**0.41** (0.01)	**0.52** (0.001)	**0.38** (0.03)	**0.49** (0.003)	**0.36** (0.04)
$\phi _{0}$ wo IPSL	0.29 (0.11)	**0.4** (0.02)	0.33 (0.07)	0.27 (0.13)	0.17 (0.36)

The second row shows the results after removing the two versions of IPSL-CM5A from the ensemble because of their outlier nature.* P*-values are in parentheses, bold values indicate* p*-values less than 0.05

### Jet latitude bias

**Fig. 10:**
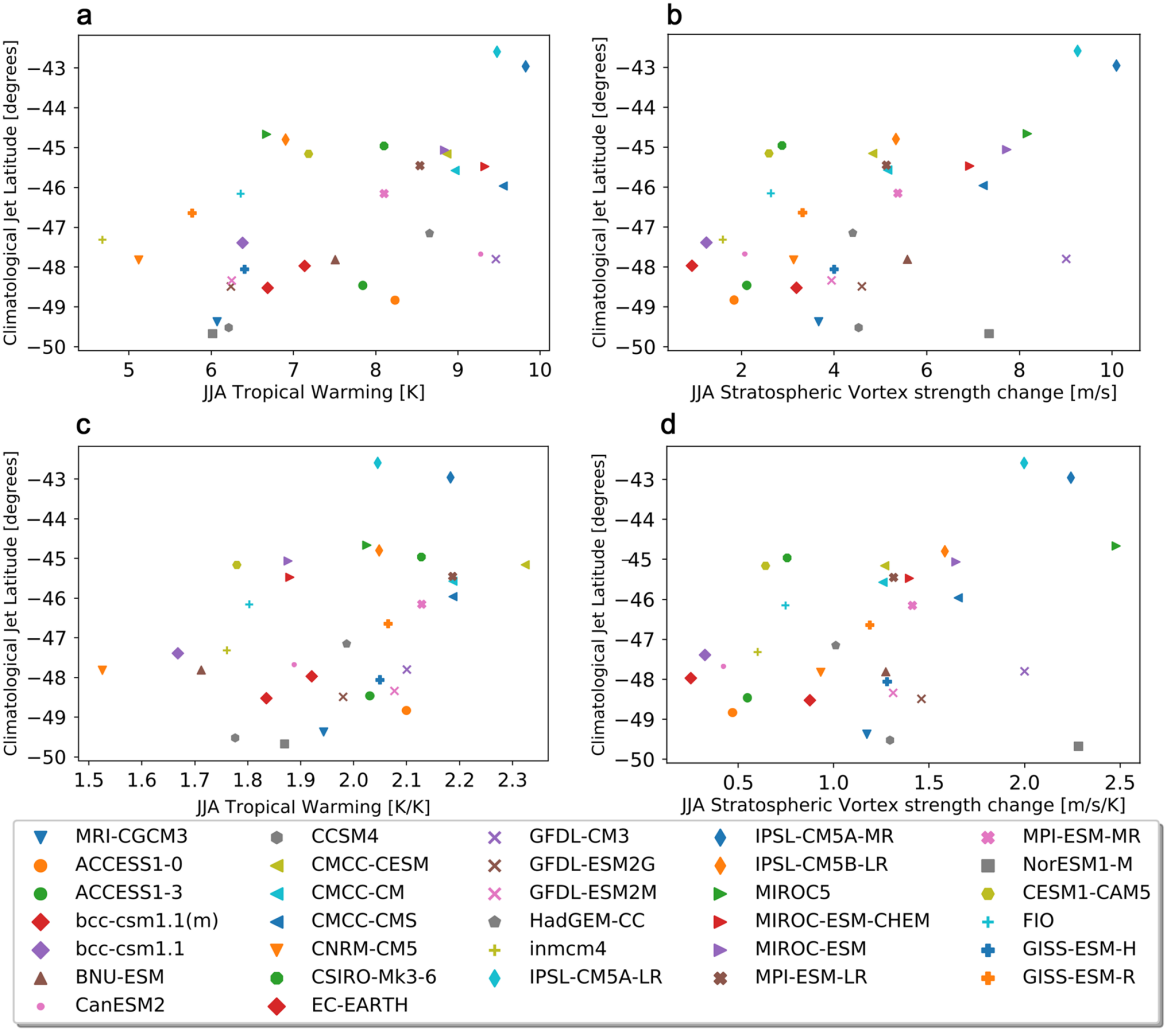
Climatological position of the midlatitude jet in the historical reference period 1940–1970 vs **a** tropical warming, **b** stratospheric vortex strengthening. **c**, **d** Are the same as (**a**–**b**) but the driver indices are scaled by global warming. The two outliers in terms of latitude bias are IPSL-CM5A-LR and IPSL-CM5A-MR

A correlation between the annual mean jet shifts in response to the RCP8.5 scenario and the climatological positions of the jet stream in the SH was identified across the CMIP3 models (Kidston and Gerber 2010). Simpson and Polvani (2016) studied this relationship in the CMIP5 model ensemble. They found that the correlation between the jet position in the historical simulations and the jet shift by the end of the century for the RCP8.5 scenario is strong for winter (JJA) but not statistically significant for summer (DJF). We similarly find a statistically significant correlation between our JJA indices and the climatological jet position (Fig. 10 and Table 3). However there are two models with outlier behaviors, namely the low and high resolution versions of IPSL-CM5A, which have an extreme equatorward jet stream bias, with the jet located at approximately $43^{\circ }\hbox {S}$. When these models are removed from the ensemble, the correlation diminishes considerably (Table 3), corroborating the outlier nature of this model (Fig. 10). Moreover, we also find a statistically significant correlation between the jet latitude bias and global warming, which Simpson and Polvani (2016) did not control for. After scaling the indices by global warming, the correlation with the jet latitude diminishes substantially (Table 3). (When regressing out global warming as in Ceppi and Shepherd (2019), rather than scaling by global warming, the correlation similarly loses statistical significance.) We conclude that the model bias in the jet position is not a confounding factor for this analysis after removing the two versions of IPSL-CM5A from the ensemble and applying the pattern scaling assumption in the regression framework. We note the correlation between the jet latitude bias and climate sensitivity as a potential confounding factor when analyzing the impacts of this bias.

**Fig. 11:**
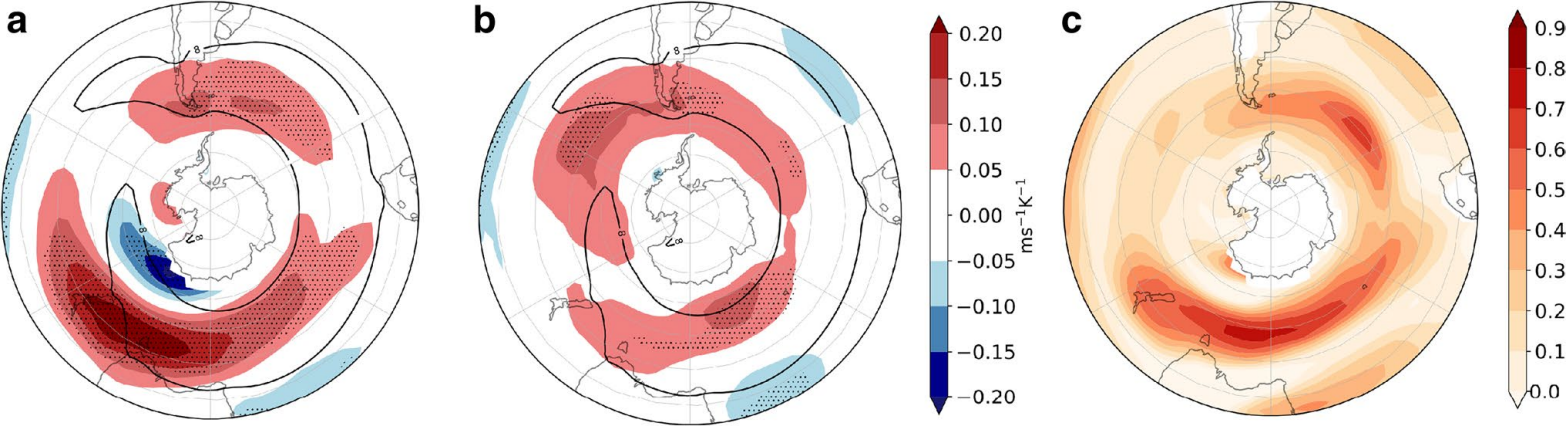
As Fig. 5 but for JJA, except the sensitivities are to the uncertainties in the JJA remote drivers **a**$\varDelta T_{trop}/\varDelta T$ and **b**$\varDelta U_{strat}/\varDelta T$, determined through sequential regressions (see “Appendix A” for mathematical details) and **c** shows the fraction of variance ($R^{2}$ coefficient) explained by the linear model (4)

### Circulation and precipitation sensitivity to remote drivers

We analyze the circulation response to the remote drivers introduced in Sect. 2.2 by applying the regression framework in Sect. 2.3 to *u*850 (Fig. 11). In contrast to DJF, the climatological mean westerly zonal winds show a marked asymmetric structure in JJA. There is a minimum in the south Pacific and a spiral structure that leads to a more poleward location of the jet to the south of Australia. Accordingly, an asymmetric pattern is also observed in the wind response to TW, characterized by a large positive wind response between the southern Indian and southwestern Pacific oceans, a positive but weaker response from South America to the southwestern Atlantic, and a negative response south of New Zealand (Fig. 11a). The latter could be related to the changes in the response of the teleconnection that typically extends between the southwestern Pacific Ocean and South America (Kidson 1988). In contrast, the circulation response to VS is more zonally extended (Fig. 11b). The magnitude of the responses is comparable between both drivers except for the strong eastward response to TW located to the south east of Australia. Locally the remote drivers explain up to $60\%$ of the variance and the regression model is particularly good at explaining the inter-model variance near the position of the jet maximum (Fig. 11c).

**Fig. 12:**
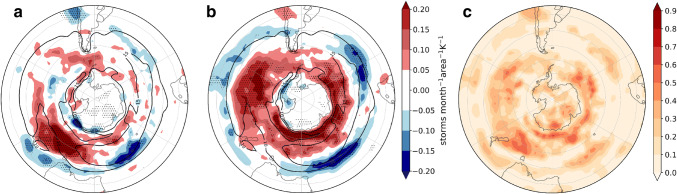
As Fig. 11 but for cyclone density. Black contours show the 10 storms month$^{-1}$ unit area$^{-1}$ MEM in the historical simulations

Mean conditions of the JJA cyclone track density (Fig. 12) exhibit, like the zonal winds, a spiral-like structure with two main storm paths along the southwest Pacific. The cyclone density response to TW is very large to the south east of Australia, like that of the zonal winds. There is a weaker cyclone density increase in the Pacific, and a cyclone density decrease to the west of Australia. On the other hand, in response to the VS there is an increase and a poleward shift of cyclone density along the subpolar latitudes with a maximum in the south Pacific and a decrease in midlatitudes with maxima to the south of South Africa and over the southeastern Indian Ocean.

**Fig. 13:**
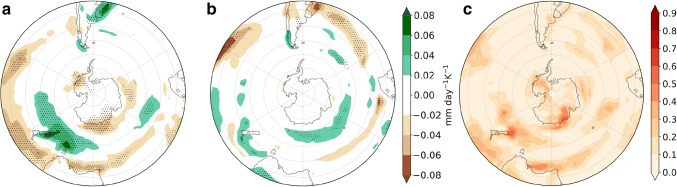
As Fig. 11 but for precipitation response (mm day$^{-1}\hbox { K}^{-1}$)

The precipitation response to TW is consistent with the cyclone density response in New Zealand, Tasmania and the south of Australia (Figs. 12a, 13a). Although in Tierra del Fuego this also seems to be the case, the precipitation response in the rest of South America is not apparently related to cyclone density. In response to VS we see enhanced precipitation to the north of New Zealand and Tierra del Fuego. Because we focus on inhabited regions, in the next section we do not analyze the precipitation changes along the Antarctic coast, although in this region TW shows wide explanatory power.

**Fig. 14:**
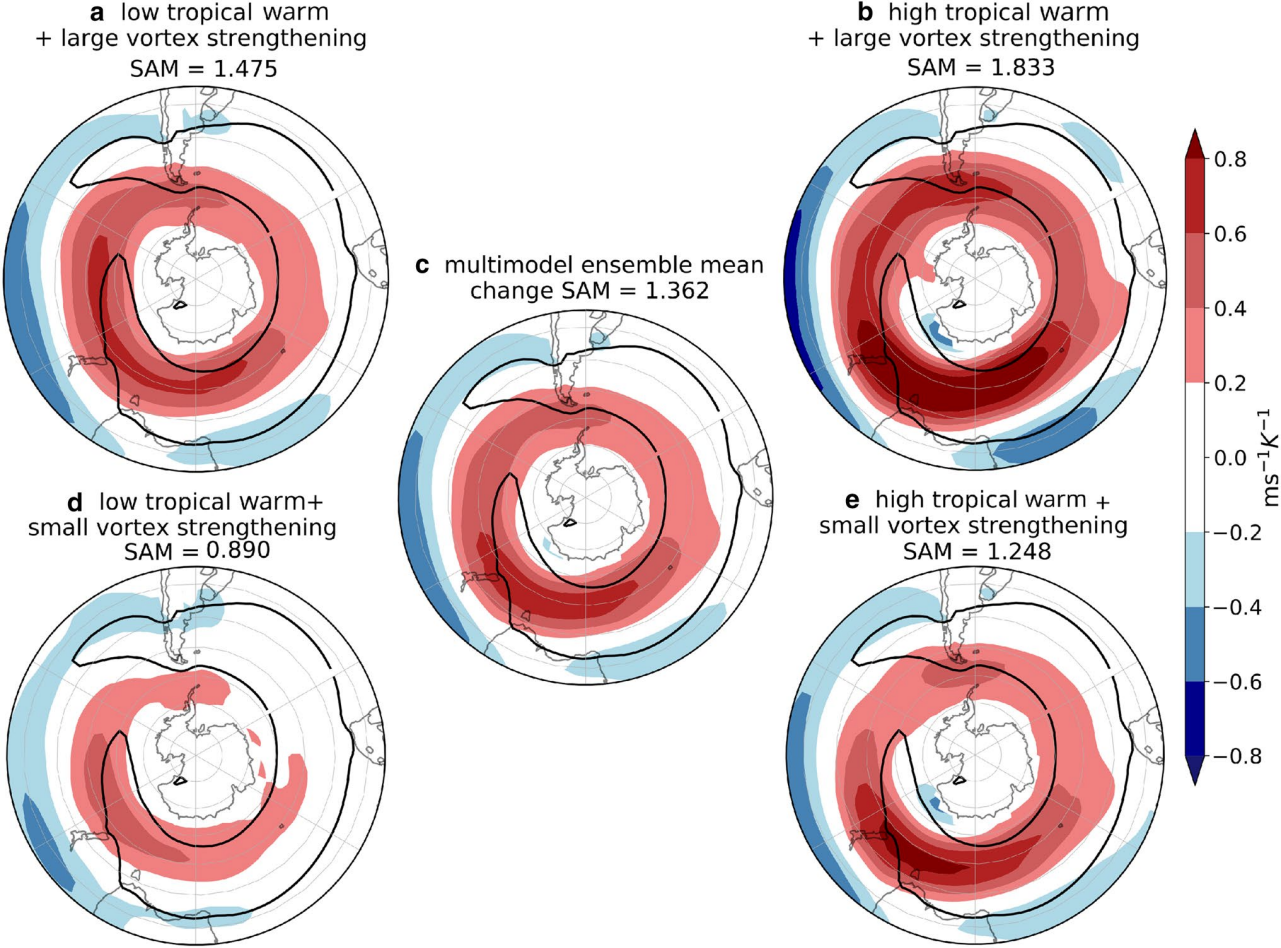
As Fig. 8 but for JJA

### Storylines of regional wind and precipitation changes

Figure 14 shows the *u*850 response maps for each of the four storylines considered, as well as the MEM. As in DJF, each of the storylines is associated with a value of the SAM index. In contrast to the case for DJF, the storylines in JJA are not located symmetrically in the ellipse space (Fig. 4b). A storyline with low TW ($\sim 1.65\hbox { KK}^{-1}$) and a small VS ($\sim 0.4\hbox { ms}^{-1}\,\hbox {K}^{-1}$) is associated with a weak strengthening of *u*850 at subpolar latitudes and small SAM index value (Fig. 14d). The response is stronger in the storyline associated with a high TW ($\sim 2.2\hbox { KK}^{-1}$) while keeping a small VS ($\sim 0.6\hbox { ms}^{-1}\,\hbox {K}^{-1}$), which leads to a much more symmetric response and a strengthening of the jet over New Zealand, shifting the westerly winds equatorward in this sector (Fig. 14e). In contrast, the storyline associated with a large VS ($\sim 1.8\hbox { ms}^{-1}\,\hbox {K}^{-1}$) and a low TW ($\sim 1.75\hbox { KK}^{-1}$) exhibits no strong equatorward shift but an even more zonally symmetric response with a maximum at the exit region of the climatological jet indicating an extension of the latter to the east (Fig. 14a). Finally, the high TW ($\sim 2.3\hbox { KK}^{-1}$) and large VS ($\sim 2.1\hbox { ms}^{-1}\,\hbox {K}^{-1}$) storyline exhibits the strongest *u*850 response at both subpolar and midlatitudes and the largest SAM index value (Fig. 14b).

**Fig. 15:**
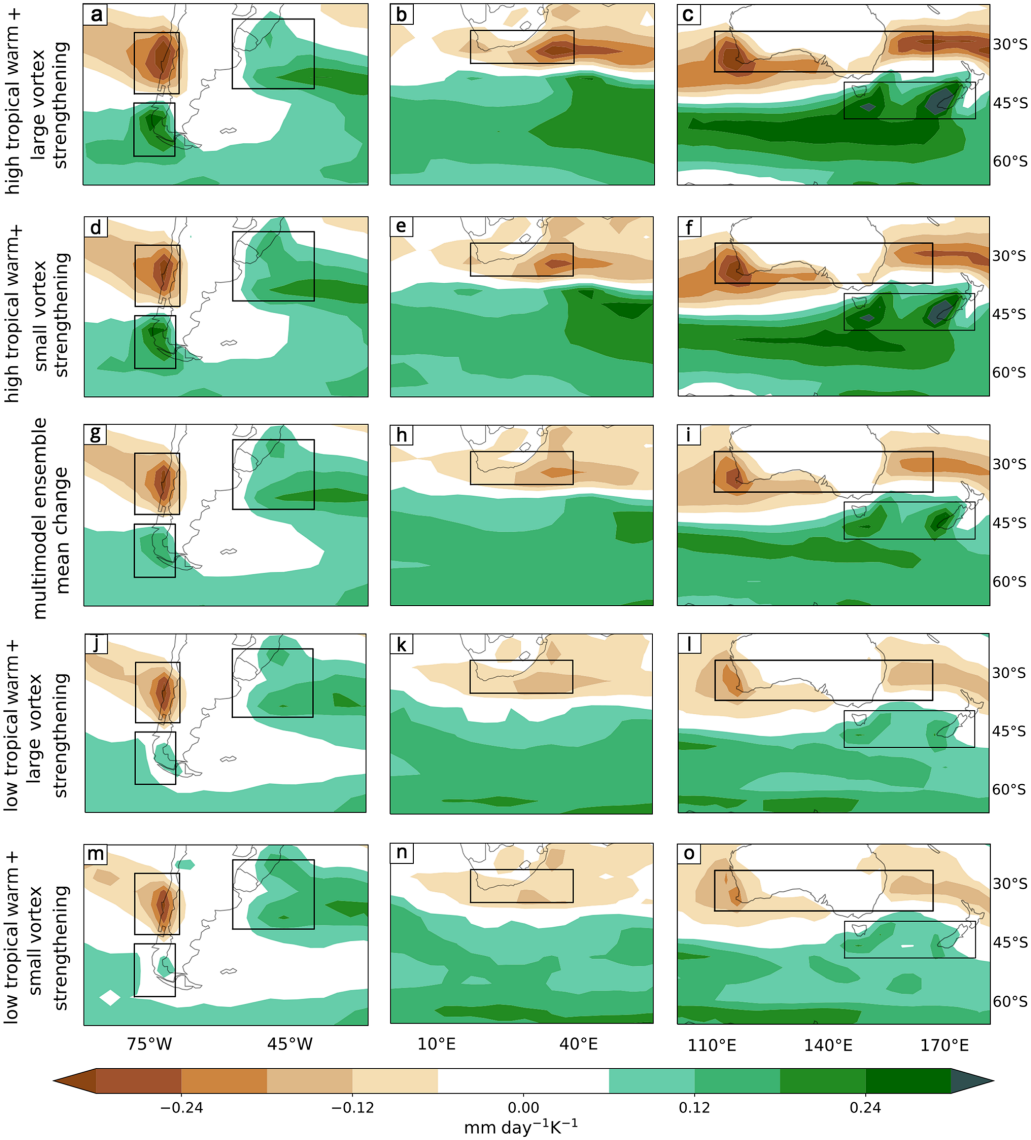
As Fig. 9 but for JJA, referencing storylines in Fig. 14

As for DJF, we show the precipitation changes related to the four storylines in Fig. 14 in the three continental domains of the SH (Fig. 15). In this season we identify six regions and, in Table 4, we present the area average contribution of each remote driver to the precipitation change per degree of warming, the precipitation change per degree of warming for all four storylines and the median absolute deviation of the area averaged residuals in the linear model (Eq. 4). As in DJF, the ordering of the area average precipitation changes between storylines is the same if the storylines are instead evaluated through model averages (Sect. 2.4), which provides a measure of robustness (not shown). As was mentioned earlier, the drying band in JJA is located more equatorward compared to its location in DJF (compare Fig. 9g–i to Fig. 15g–i). Correspondingly, drying responses are observed across the southern portions of South Africa and Australia, in contrast to the wetting seen over these regions in DJF, and the drying region on the western coast of South America is located further north than in DJF. Consistent with the drying band being located more equatorward in JJA, the wetting band is located more equatorward as well. All storylines show a wetting across the entire hemisphere to the south of $40^{\circ }\hbox {S}$.

**Table 4:** Area average of JJA precipitation changes (mm day $^{-1}\hbox { K}^{-1}$) associated with each remote driver, and in the four storylines shown in Fig. 15, together with the median absolute deviation (MAD) of the residuals from the statistical model (Eq. 4)

JJA							
Region	TW	VS	Low TW Small VS	High TW Small VS	Low TW Large VS	High TW Large VS	Residual MAD
Subtropical Andes	− 0.022	0.005	− 0.13	− 0.19	− 0.12	− 0.18	0.07
Tierra del Fuego	0.015	0.024	0.04	0.08	0.10	0.15	0.06
Southeastern South America	0.020	− 0.013	0.08	0.12	0.05	0.10	0.04
South of South Africa	− 0.007	− 0.004	− 0.10	− 0.12	− 0.11	− 0.13	0.04
South of Australia	− 0.021	0.009	− 0.07	− 0.12	−0.05	− 0.11	0.04
Tasmania and NZ	0.039	0.007	0.06	0.17	0.10	0.20	0.06

As in DJF, the SAM index is approximately equally sensitive to both drivers and is most extreme in the “High TW-Large VS” storyline, but the precipitation changes over land respond differently to the drivers depending on the region, and are not well explained by the SAM changes (Table 4). In Australasia the main sensitivity is to TW, leading to wetting in Tasmania/NZ and to drying in South of Australia. On the western side of South America the two drivers are of roughly equal importance and act in concert, thus coherently with the SAM, to induce wetting in Tierra del Fuego, however they act in opposite directions in the Subtropical Andes. What we observe in the Subtropical Andes is consistent with Seager et al. (2019), who find that the interannual precipitation variability of SH mediterranean regions like the Subtropical Andes is not strongly related to the SAM. In Southeastern South America the drivers likewise act in opposite directions, and in an opposite sense than in the Subtropical Andes. Thus the most extreme precipitation changes are sometimes found in the intermediate storylines (Fig. 15d–f, j–l).

## Summary by region

### DJF precipitation changes

*Extratropical Andes* This is a wet region. The MEM projects drying over the region (Fig. 9g). The TW is the main contributor to drying in this region (Table 2), and the “High TW-Late VB” storyline (Fig. 9a) provides the largest drying, the “Low TW-Early VB” storyline (Fig. 9m) provides the smallest drying, and the intermediate storylines provide intermediate levels of drying. The difference between the most extreme storylines is large compared to the unexplained variability (Table 2).

*Southeastern South America* This is a wet region and the precipitation mechanisms are diverse, as the region is affected by tropical climate patterns, SAM phases, cold fronts and local convection. The MEM projects a wetting (Fig. 9g). In this region the TW acts in the opposite sense to the VB and both seem to be important (Table 2), but the VB is related to larger changes. The highest wetting is related to the “Low TW-Late VB” storyline (Fig. 9d). Since the SAM response to TW and VB has the same sign, this shows that precipitation changes in this region are not well characterized by SAM changes.

*East of South Africa* In DJF, this is the wet region of South Africa. Precipitation here is related to moisture convergence in the South Indian Convergence Zone. A wetting is projected by the MEM (Fig. 9h). In this region the TW acts in the opposite sense to the VB, so the same comments apply as in Southeastern South America. The highest wetting is related to the “Low TW-Late VB” storyline (Fig. 9e). However, the unexplained variability is particularly high in this region (Table 2).

*South East of Australia* This is a wet region in DJF. Enhanced precipitation is projected by the MEM (Fig. 9i). As for Southeastern South America and East of South Africa, the wetting is clearly linked with an enhanced storm density, which responds to the VB delay (Fig. 6b), and the two drivers act in the opposite sense and are both equally important. The largest wetting is provided by the “Low TW-Late VB” storyline (Fig. 9f).

*Tasmania* This region does not have a dry season. Nevertheless, DJF is the driest of the year. A drying is projected by the MEM over this region (Fig. 9i). The TW is the main driver of the precipitation changes with a smaller and opposing role for the VB (Table 2). The largest drying is provided by the “High TW-Early VB” storyline (Fig. 9l).

### JJA precipitation changes

*Subtropical Andes* This is a wet region, where precipitation is caused by frontal activity favoured by midlatitude westerly winds. A robust drying is projected by the MEM (Fig. 15g). All storylines show a high level of drying. However the two drivers act in opposite directions and the TW is the most important driver of drying (Table 4), so that the precipitation response is not proportional to the SAM response. The most extreme drying is provided by the “High TW-Small VS” storyline (Fig. 15d), although the response is almost equal to that of the “High TW-Large VS” storyline (Fig. 15a).

*Tierra del Fuego* This is a wet region. A wetting is projected by the MEM (Fig. 15g). Both drivers are important and induce changes in the same sense. Therefore, the largest wetting is provided by the “High TW-Large VS” storyline and the weakest wetting by the “Low TW-Small VS” storyline (Fig. 15a, m). Intermediate storylines show intermediate responses. Thus, in this region the magnitude of the precipitation changes associated with each storyline is related to the intensity of the SAM change.

*Southeastern South America* This is a wet region. A robust wetting is projected by the MEM (Fig. 15g). As in DJF, the responses to the two drivers act in the opposite sense. The most extreme wetting is provided by the “High TW-Small VS” storyline (Fig. 15d), while there is a low wetting in the “Low TW-Large VS” storyline (Table 4, Fig. 15j). As in DJF, the circulation and precipitation changes in this region are not well characterized by SAM changes.

*South of South Africa* The west tip of South Africa, contained within this large region, is the wet region of South Africa in JJA. The MEM projects drying across the region (Fig. 15h). Both drivers are of comparable importance and contribute to drying, therefore the largest drying is provided by the “High TW-Large VS” storyline (Fig. 15b) and the smallest drying by the “Low TW-Small VS” storyline (Fig. 15n). However, the differences are not particularly large compared to the unexplained variability or to the MEM.

*South of Australia* JJA is the wet season for most of this region. The region is projected to dry in the MEM (Fig. 15i). The TW is the most important driver of drying in this region. This is reflected in the fact that for the same VS, storylines show drier conditions if the TW is high (Table 4), but there is almost no sensitivity to VS (Fig. 13b). Since the SAM is affected by both drivers, this means that precipitation changes are not only related to SAM changes in this region.

*Tasmania and New Zealand* JJA is the wet season for these regions, where the west coasts of both Tasmania and New Zealand are affected by cold front activity. The MEM projects wetting in this region (Fig. 15i). The TW is the most important driver of wetting in this region, while the VS has a negligible role. The most extreme wetting is provided by the “High TW-Large VS” storyline (Fig. 15c).

## Discussion and conclusions

In this study we have constructed storylines of the Southern Hemisphere circulation and precipitation response to greenhouse gas forcing during austral summer and winter based on the strength of the tropical upper-tropospheric warming and the stratospheric polar vortex response, conditional on the global-mean warming level. The uncertainty in these two remote drivers for a given global-mean warming may be regarded as an epistemic uncertainty (Shepherd 2019), which may be reduced in the future as a better physical understanding of the cause of these driver responses is obtained. In this way, future research may eliminate some of the storylines described here. In the meantime, the different storylines provide plausible manifestations of change at the regional scale, which could be used for a regional risk assessment. It should be noted that individual model responses are not always consistent with the expectation from the storylines. Thus, the explanatory power of the storylines applies only to their description of the entire set of CMIP5 models considered, and is not deterministic for particular models. This must be borne in mind when choosing particular GCMs to drive Regional Climate Models.

The main results of this paper can be summarized as follows:

- While the response to tropical warming (TW) leads to a strengthening of the SH westerly winds at 850 hPa, the response to a delayed breakdown (for DJF) or strengthening (for JJA) of the stratospheric vortex (VB delay and VS, respectively) is a poleward shift of the westerly winds.
- The SAM index responds to both drivers with the same sign and comparable amplitude in both seasons. As a result, the storyline describing the most extreme positive SAM change is found for a high tropical warming and a large strengthening/delay in the stratospheric vortex.
- However, the response of the SAM is not sufficient to characterise regional climate change, since regional circulation and precipitation over the examined land regions does not always respond equally, or even with the same sign, to the two drivers. For example, in DJF TW generally leads to drying and VB delay to wetting, even though the sign of the SAM response is positive in both cases.
- As a consequence of the above, the two drivers have significant explanatory power in different regions and tailored regional storylines must be considered. In some regions, namely, Southeastern South America (DJF and JJA), East of South Africa, East of Australia and Tasmania (DJF), South of South Africa and Tierra del Fuego (JJA), the precipitation change within each storyline depends on the combined climate response of the two drivers, but in other regions, namely, Extratropical and Subtropical Andes (DJF and JJA respectively), South of Australia, Tasmania and New Zealand (JJA), the main difference between storylines can be attributed to the response of just one remote driver (see Tables 2, 4).

In light of the relationship between the midlatitude jet bias and the jet shift identified for JJA by Simpson and Polvani (2016), we examined the role of the jet bias as a potentially confounding factor in our analysis. We found a strong correlation between jet bias and global warming (i.e. climate sensitivity). However, this correlation mainly arises from the inclusion of two versions of the same model, IPSL-CM5A-LR and IPSL-CM5A-MR, which both have extreme jet biases. We thus removed these models from our JJA analysis. After scaling by global-mean warming, the relation between jet bias and driver response is not statistically significant.

These results are based on the assumption that the changes in all fields and remote drivers scale linearly with climate sensitivity. Although pattern scaling has been shown to be a useful approximation (Zappa and Shepherd 2017; Zelazowski et al. 2018), it can certainly be improved (Tebaldi and Arblaster 2014; Herger et al. 2015). For example, it has been shown that the circulation response can be sensitive to the rate of $\hbox {CO}_{2}$ emissions or aerosol radiative responses independently from global warming (Grise and Polvani 2014) and that the stratospheric vortex also exhibits a weaker “direct” response to greenhouse gas forcing (Ceppi and Shepherd 2019). However, these effects are not expected to be a limitation for the study performed here, given the focus on SH midlatitudes at a fixed time horizon under the same forcing scenario.

By defining the climate response as the difference between the 1940–1970 climatology in the historical simulation (1950–1980 for cyclone density) and 2069–2099 in the RPC8.5 simulation, we deliberately exclude the effect of the ozone hole, which began to emerge in the mid-1970s. Ozone depletion is not relevant for JJA (McLandress et al. 2011), but can be expected to have an impact on the DJF circulation and precipitation, since stratospheric ozone depletion induces local radiative cooling which leads to a strengthening of the vortex and a delay in the vortex breakdown (McLandress et al. 2011; Sun et al. 2014; Screen et al. 2018). However, the effect of ozone depletion on the vortex breakdown is expected to be small by the end of the century (McLandress et al. 2010). With our approach we thus isolate the changes driven by greenhouse gas forcing from those induced by ozone depletion.

Perhaps the most far-reaching aspect of our results is that the tropical and high-latitude drivers of circulation change project quite differently onto the mid-latitude westerlies, and thus onto precipitation changes. In that respect, the concept of a ‘tug of war’ between tropical and high-latitude drivers may be overly simplified. For example, Southeastern South America has an opposite response to the two drivers in both seasons; hence the most extreme storylines of regional climate change correspond to intermediate storylines in terms of the SAM. This point is also made by Baker et al. (2017), who distinguished the shifting and strengthening of the jet as distinct responses to different thermal forcings in an idealized model. Although using EOF1 (latitude shift) and EOF2 (strengthening) (e.g. Boljka et al. (2018)) could potentially capture these two jet responses, we would argue that the annular modes of variability are merely descriptors rather than drivers of circulation and storm track changes. Moreover they characterize only the zonal mean behavior. In any case, SAM indices defined as the EOF1 (mainly related to the latitudinal shift of the winds) may capture only a fraction of the future circulation and precipitation changes.

In both seasons the zonal wind sensitivity to the tropical warming has a gap between $110^{\circ }\hbox {W}$ and $70^{\circ }\hbox {W}$. This sector is affected on interannual to multi-decadal timescales by Rossby wave trains from the tropical oceans which can either be reinforced or inhibited by the SAM (Silvestri and Vera 2009). The fraction of zonal wind variance explained in both DJF and JJA also shows a clear gap in this sector. Including a remote driver to capture the influence of tropical asymmetric forcing such as SST patterns could potentially explain a larger fraction of the inter-model variance in the circulation response and hence lead to the construction of more comprehensive storylines.

## Appendix A

*DJF storyline evaluation* We analyze four storylines for each season. The storylines are characterized by combinations of extreme values of the remote drivers compared to the multimodel ensemble mean value. For this, each storyline is evaluated such that the remote drivers have the same amplitude in the standardized space and they lie on the edge of the $80\%$ confidence region of their joint distribution. This means they are evaluated where the lines $(\varDelta T_{trop}/\varDelta T)^{\prime } = (VB_{delay}/\varDelta T)^{\prime }$ and $(\varDelta T_{trop}/\varDelta T)^{\prime } = -(VB_{delay}/\varDelta T)^{\prime }$ intercept the ellipse of $80\%$ confidence (Fig. 4a). The storyline coefficient can be worked out by the intersection of these lines with the ellipse equation. In the case of DJF, the correlation between the indices is almost null, so the confidence region can be well approximated by the ellipse (circle) with the form

A1 $$\begin{aligned} \left[ \left( \frac{\varDelta T_{trop}}{\varDelta T} \right) ^{\prime }\right] ^{2} + \left[ \left( \frac{VB_{delay}}{\varDelta T} \right) ^{\prime }\right] ^{2} = \chi ^{2}(0.8,2), \end{aligned}$$

and the storyline coefficient takes the value

A2 $$\begin{aligned} t_{s} = \sqrt{\chi ^{2}(0.8,2)/2} \approx 1.26. \end{aligned}$$

The response pattern for a given field can then be evaluated as

A3 $$\begin{aligned} \frac{\varDelta C_{x}}{\varDelta T} = \hat{a}_{x} \pm \hat{b}_{x}t_{s} \pm \hat{c}_{x}t_{s}, \end{aligned}$$

for the High TW—Late VB and the Low TW—Early VB storylines, and

A4 $$\begin{aligned} \frac{\varDelta C_{x}}{\varDelta T} = \hat{a}_{x} \pm \hat{b}_{x}t_{s} \mp \hat{c}_{x}t_{s}. \end{aligned}$$

for the intermediate storylines.

*JJA storyline evaluation* As for DJF, the pattern of response of a field in JJA can be modeled as in (4). However, because of the correlation between the drivers, to compute the sensitivities to the remote drivers while controlling for the influence of the other driver, we apply the sequential regressions

A5 $$\begin{aligned} \frac{\varDelta C_{xm}}{\varDelta T_{m}}=\, & {} a_{x} + c_{x}^{*} \left( \frac{\varDelta U_{strat}}{\varDelta T}\right) _{m}' + e_{xm}^{*}, \end{aligned}$$

A6 $$\begin{aligned} e_{xm}^{*}=\, & {} b_{x}\left( \frac{\varDelta T_{trop}}{\varDelta T}\right) _{m}' + e_{xm}, \end{aligned}$$

and

A7 $$\begin{aligned} \frac{\varDelta C_{xm}}{\varDelta T_{m}}=\, & {} a_{x} + b_{x}^{*} \left( \frac{\varDelta T_{trop}}{\varDelta T}\right) _{m}' + e_{xm}^{*}, \end{aligned}$$

A8 $$\begin{aligned} e_{xm}^{*}=\, & {} c_{x}\left( \frac{\varDelta U_{strat}}{\varDelta T}\right) _{m}' + e_{xm}. \end{aligned}$$

Note that $a_{x}$ is the multimodel ensemble mean. The coefficients $c_{x}^{*}$ and $b_{x}^{*}$ quantify the sensitivity of the regional response to the anomalies in the remote drivers $\varDelta U_{strat}/\varDelta T$ and $\varDelta T_{trop}/\varDelta T$, respectively, while $b_{x}$ and $c_{x}$ quantify the sensitivity of the regional response to the anomalies in the remote drivers $\varDelta T_{trop}/\varDelta T$ and $\varDelta U_{strat}/\varDelta T$ having previously controlled for the other remote driver.

The $80\%$ confidence region of a joint distribution for two correlated normally distributed variables is defined by the ellipse with the form

A9 $$\begin{aligned}&\left[ \left( \frac{\varDelta T_{trop}}{\varDelta T} \right) ^{\prime }\right] ^{2} - 2r\left( \frac{\varDelta T_{trop}}{\varDelta T} \right) ^{\prime }\left( \frac{\varDelta U_{strat}}{\varDelta T} \right) ^{\prime } \nonumber \\&\quad + \left[ \left( \frac{\varDelta U_{strat}}{\varDelta T} \right) ^{\prime }\right] ^{2} = (1-r^{2})c, \end{aligned}$$

where *r* is the correlation coefficient, in this case $r \approx 0.27$ and $\hbox {c} = \chi ^{2}(0.8,2)$.

If we select the storylines so that they have the same amplitude in the standardized space, they are evaluated where the lines $(\varDelta T_{trop}/\varDelta T)^{\prime } = (\varDelta U_{strat}/\varDelta T)^{\prime }$ and $(\varDelta T_{trop}\varDelta T)^{\prime } = -(\varDelta U_{strat}/\varDelta T)^{\prime }$ intercept the confidence ellipse. The storyline coefficient can be worked out by the intersection of these lines with the ellipse

A10 $$\begin{aligned} t^{2} - 2rt^{2} + t^{2} = (1-r^{2})c, \end{aligned}$$

so

A11 $$\begin{aligned} t_{s_{1}} = \sqrt{\frac{(1-r^{2})}{2(1 - r)}c}, \end{aligned}$$

and

A12 $$\begin{aligned} t^{2} + 2rt^{2} + t^{2} = (1-r^{2})c, \end{aligned}$$

so

A13 $$\begin{aligned} t_{s_{2}} = \sqrt{\frac{(1-r^{2})}{2(1 + r)}c}. \end{aligned}$$

Finally the response pattern for a given field is evaluated as

A14 $$\begin{aligned} \frac{\varDelta C_{x}}{\varDelta T} = \hat{a}_{x} \pm \hat{b}_{x}t_{s1} \pm \hat{c}_{x}t_{s1} \end{aligned}$$

where

A15 $$\begin{aligned} t_{s_{1}} = \sqrt{\frac{(1-r^{2})}{2(1-r)}\chi ^{2}(0.8,2)} \approx 1.41 \end{aligned}$$

for the High TW - Large VS and Low TW - Small VS storylines, and

A16 $$\begin{aligned} \frac{\varDelta C_{x}}{\varDelta T} = \hat{a}_{x} \pm \hat{b}_{x}t_{s2} \mp \hat{c}_{x}t_{s2}, \end{aligned}$$

where

A17 $$\begin{aligned} t_{s_{2}} = \sqrt{\frac{(1-r^{2})}{2(1+r)}\chi ^{2}(0.8,2)} \approx 1.07 \end{aligned}$$

for the intermediate storylines.

All the regressions are computed independently for each grid point and each coefficient is computed together with its corresponding p value (according to a two-tailed Student’s t distribution). Panels a and b in Figs. 5, 6 and 7 show the coefficients $\hat{b}_{x}$ and $\hat{c}_{x}$ computed with the regression framework applied to three DJF fields, namely zonal-wind, cyclone density and precipitation. The same is shown in Figs. 11, 12 and 13 for JJA fields. Stippling in these figures indicates grid points for which the coefficient has a* p* value < 0.05, which is chosen as the significance level.

## Appendix B

*Confidence intervals for remote drivers* In Fig. 4 we show, for each model, the values of the remote drivers’ indices with their corresponding error bars. We here provide detail on how the confidence intervals were computed. We do not find a detectable lag-1 autocorrelation^1^ in the year-to-year internal variability of the drivers during the reference periods, hence we treat it as white noise. Defining $\beta$ as the response (i.e., the difference between a metric in the RCP8.5 and the historical simulations), we know that the difference between two t-distributions is approximately normally distributed. Hence, the confidence interval is evaluated as $(\overline{\beta } - 1.96SE_{\beta }, \overline{\beta } + 1.96SE_{\beta })$ where the standard error ($SE_{\beta }$) is:

B.1 $$\begin{aligned} SE_{\beta }=\, & {} \sqrt{{SE_{hist}}^{2} + {SE_{RCP8.5}}^{2}}, \end{aligned}$$

B.2 $$\begin{aligned} SE_{hist}=\, & {} \frac{\sigma _{hist}}{\sqrt{N_{hist}ENS_{hist}}} \end{aligned}$$

and

B.3 $$\begin{aligned} SE_{RCP8.5} = \frac{\sigma _{RCP8.5}}{\sqrt{N_{RCP8.5}ENS_{RCP8.5}}}. \end{aligned}$$

$\sigma _{hist}$ and $\sigma _{RCP8.5}$ are the inter-annual standard deviations of the detrended time series. $N_{hist}$ and $N_{RCP8.5}$ are the number of years analyzed (31). $ENS_{hist}$ and $ENS_{RCP8.5}$ are the number of ensemble members considered for each simulation (see Table 1).
